# Elongation factor TFIIS is essential for heat stress adaptation in plants

**DOI:** 10.1093/nar/gkac020

**Published:** 2022-01-31

**Authors:** István Szádeczky-Kardoss, Henrik Mihály Szaker, Radhika Verma, Éva Darkó, Aladár Pettkó-Szandtner, Dániel Silhavy, Tibor Csorba

**Affiliations:** Genetics and Biotechnology Institute, MATE University, Szent-Györgyi A. u. 4, 2100 Gödöllő, Hungary; Genetics and Biotechnology Institute, MATE University, Szent-Györgyi A. u. 4, 2100 Gödöllő, Hungary; Faculty of Natural Sciences, Eötvös Lóránd University, Pázmány Péter sétány 1/A, 1117 Budapest, Hungary; Institute of Plant Biology, Biological Research Centre, Temesvári krt. 62., 6726 Szeged, Hungary; Genetics and Biotechnology Institute, MATE University, Szent-Györgyi A. u. 4, 2100 Gödöllő, Hungary; Doctorate School of Biological Sciences, MATE University, Pater Karoly u. 1, 2100 Gödöllő, Hungary; Agricultural Institute, Centre for Agricultural Research, Brunszvik u. 2., 2462 Martonvásár, Hungary; Proteomics Laboratory, Biological Research Centre, Temesvári krt. 62., 6726 Szeged, Hungary; Institute of Plant Biology, Biological Research Centre, Temesvári krt. 62., 6726 Szeged, Hungary; Genetics and Biotechnology Institute, MATE University, Szent-Györgyi A. u. 4, 2100 Gödöllő, Hungary

## Abstract

Elongation factor TFIIS (transcription factor IIS) is structurally and biochemically probably the best characterized elongation cofactor of RNA polymerase II. However, little is known about TFIIS regulation or its roles during stress responses. Here, we show that, although TFIIS seems unnecessary under optimal conditions in *Arabidopsis*, its absence renders plants supersensitive to heat; *tfIIs* mutants die even when exposed to sublethal high temperature. TFIIS activity is required for thermal adaptation throughout the whole life cycle of plants, ensuring both survival and reproductive success. By employing a transcriptome analysis, we unravel that the absence of TFIIS makes transcriptional reprogramming sluggish, and affects expression and alternative splicing pattern of hundreds of heat-regulated transcripts. Transcriptome changes indirectly cause proteotoxic stress and deterioration of cellular pathways, including photosynthesis, which finally leads to lethality. Contrary to expectations of being constantly present to support transcription, we show that TFIIS is dynamically regulated. TFIIS accumulation during heat occurs in evolutionary distant species, including the unicellular alga *Chlamydomonas reinhardtii*, dicot *Brassica napus* and monocot *Hordeum vulgare*, suggesting that the vital role of TFIIS in stress adaptation of plants is conserved.

## INTRODUCTION

Environmental changes continuously affect growth and reproduction of organisms. Plants have sophisticated mechanisms for the adaptation to external factors and for balancing their development and stress responses accordingly. Transcriptional regulation plays a key role in stress responses. Traditionally, it was considered that transcription is regulated at the initiation step. However, recent results have shown that elongation is also precisely regulated (1–3).

In eukaryotes, RNA polymerase II (RNAPII) is responsible for transcription of most mRNAs. Diverse circumstances may halt and cause subsequent backtracking of RNAPII. During backtracking, the 3′-end of the nascent RNA is displaced from the polymerase active core. To resume transcription, the cleavage of the protruding 3′-end and re-establishment of the correct base pairing between the nascent RNA and template DNA are needed at the polymerization site. RNAPII core possesses a weak nuclease activity, which is able to perform the cleavage (4). Transcription factor IIS (TFIIS) is an important cofactor of RNAPII that assists this process: it reaches through a pore inside RNAPII in the close proximity of the active site and boosts the weak nuclease activity of the polymerase (4–8). Additionally, TFIIS binding further induces structural changes of RNAPII complex to aid reactivation following transcriptional arrests (4,9–14). TFIIS protein contains three well-defined domains: the N-terminal domain I (IIS-N) is responsible for nuclear localization and interaction with polymerase complex cofactors and regulators; the middle domain II (IIS-M) along with the linker (L) region between domains II and III is required for binding and recruitment of core RNAPII; and the most conserved C-terminal domain III (IIS-C) is responsible for nuclease activity enhancement of RNAPII.

In *Arabidopsis thaliana*, TFIIS is a single-copy gene, which is expressed throughout the whole plant body (5). Within cells, GFP–TFIIS fusion protein is localized to the nucleus, consistent with its transcriptional regulatory role. Under standard conditions, TFIIS activity seems negligible in *A. thaliana* plants, suggesting that RNAPII or alternative pathways can cope with arrests and backtracking events. In the *tfIIs* T-DNA null mutant lines, a minor number (2–3%) of genes are differentially expressed, and accordingly these plants display minor developmental phenotypes (5). The ectopic expression of a dominant negative form of TFIIS (*TFIISmut*), however, causes massive growth defects. Marked transcriptome changes are observed upon transient expression of *TFIISmut* in the *tfIIs-1* mutant background (6) and phenotype alteration in the stable *TFIISmut;*Col-0 wild-type background (15). Stable transgenic *TFIISmut;tfIIs-1* line could not be produced, probably due to lethality (6,15). These observations testify that RNAPII arrest is frequent in plants, but also that correct transcriptional output can occur properly in the absence of TFIIS under optimal conditions. TFIIS also regulates RNAPII activity in alternative ways. Significant changes in alternatively spliced mRNA isoform abundance were observed in *tfIIs-1* mutant or *TFIISmut* overexpressor *A. thaliana* plants (15,16). The loss of TFIIS had a major effect on the splicing of a reporter gene in yeast as well (17).

Under adverse conditions, error rate of transcription is increased; thus, requirement for TFIIS could be more pronounced. Indeed, in *Saccharomyces cerevisiae*, TFIIS is dispensable for growth under normal conditions but becomes vital under nucleotide-depleted conditions (18). Similarly, *A. thaliana rdo2/tfiis-2* null mutant does not display developmental alterations but becomes hypersensitive to damaging ultraviolet radiation (19). Rescue of backtracked RNAPII complexes by TFIIS in human cells was needed to ensure a proper metabolic response during hypoxic stress (14). In summary, several observations indicate that under suboptimal or adverse conditions TFIIS is required for correct transcriptome output, and thus for proper stress adaptation.

Heat stress (HS) is one of the major abiotic stresses affecting the productivity of crop plants worldwide (20–22). Plants have specific but overlapping branches of heat stress responses (HSRs) to deal with the different aspects of elevated temperature stress (23). These branches, including basal thermotolerance (BT), short and long acquired thermotolerance (SAT and LAT) and thermotolerance to moderately high temperatures (TMHT), rely on both specific and common factors to enable stress management and survival (23).

The transcriptional regulators of HSR are the heat stress factors (HSFs) (24,25). In *Arabidopsis*, the ‘master’ regulator of HSR is the HsfA1 (*HsfA1a*, *b*, *d* and *e*) transcription factor family (24) that coordinates multiple transcriptional cascades (25–32). Transcriptional activation of downstream targets genes by HSFA1s is an essential element of BT, SAT and TMHT (23). AtHsfA2 is specifically required for amplification of HS response during SAT and especially during LAT (33–36). Other HSFs have partially overlapping and/or specific functions and regulate transcriptional cascades during different stresses or stress combinations (24).

Activity of HSFs is regulated at multiple levels, including post-translational modifications, subcellular localization, oligomerization and interaction with other HSFs and heat shock proteins (HSPs) (24,25,37–39). Activated HSFs bind to *cis* elements known as the heat shock elements (HSEs) on the genome to regulate target genes (26,40–42). The simplistic chaperone titration model suggests that under normal conditions HSFs are bound and inactivated by HSPs; however, upon heat stress, denatured proteins accumulate and sequester HSPs. Consequently, HSFs are liberated to form oligomers and translocated to the nucleus where they activate target genes, including HSPs (43,44). Abundant production of diverse classes of HSPs during HSR is required for maintaining the proper cellular proteostasis (39,43). The different classes of HSPs function cooperatively. HSP70 inhibits transactivation capacity, while HSP90 blocks HSF oligomer formation and DNA binding (43,44). Small HSPs (sHSPs) act as ‘holdase’ chaperones to obstruct irreversible protein inactivation and aggregation (45–47). The *Arabidopsis* HSP101 or yeast homologue HSP104 interacts with HSP70 and its cofactors to help refolding or decay of denatured proteins (47–49). Parallel to the activation of HSR, alteration of diverse growth/development-linked pathways also occurs under acute stress conditions to help maintain homeostasis and conserve cellular energy (50).

In the present work, we show that the TFIIS elongation factor is a central gear of thermal adaptation, and it is required for viability and reproductive success throughout the full life cycle of *A. thaliana*.

## MATERIALS AND METHODS

### Plant materials

*Arabidopsis* seeds were bleach sterilized, stratified for 2 days in dark and then plated on Murashige and Skoog (Duchefa M0222, https://www.duchefa-biochemie.com) medium agar plates (0.5× Murashige and Skoog salts, 1% agar, pH 5.7). Plants were routinely grown in a Sanyo MLR-350 growth cabinet under cool white light at 21°C long-day condition (16 h light/8 h dark). Mutant seeds were ordered from NASC.

### Genotyping

Genomic DNA was extracted with extraction buffer (100 mM glycine, 10 mM EDTA, 100 mM NaCl, 2% SDS) at room temperature, purified with phenol–chloroform–isoamyl alcohol (25:24:1, pH 8.0), precipitated in ethanol and resuspended in sterile water. Genotyping PCR was done using DNA Taq polymerase (NEB, M0273S) based on the manufacturer’s instructions. For primer sequences, see Supplementary Table S1.

### Mutant and transgenic lines used in the study

tfIIs-1 (SALK_056755) and tfiis-2 (SALK_027259) (5), QK (51), pTFIIS::GSy-TFIIS;tfIIs-1, p35S::GSy-TFIIS;tfIIs-1 and p35S::Mycx2-TFIIS;tfIIs-1 (this study).

### Transgene constructs

For complementation, we prepared *pTFIIS::GSy-TFIIS*, *p35S::GSy-TFIIS* and *p35S::Mycx2-TFIIS* constructs. For the *pTFIIS::GSy-TFIIS* clone, the TFIIS gene promoter region (−572 bp upstream until ATG), the GSy tag (52), TFIIS coding region (including intron) and terminator region were PCR amplified using Phusion enzyme (Thermo Fisher) and fused together using a Gibson assembly reaction (NEB). The pTFIIS::GSy-TFIIS was cloned into pCAMBIA1301 vector at EcoRI and BstEII restriction sites using T4 DNA ligase (NEB). For the *p35S::GSy-TFIIS* construct, the GSy-TFIIS amplicon was ligated into pCAMBIA1301 at NcoI and BstEII sites. For the *p35S::Mycx2-TFIIS* construct, double Myc tag was fused to TFIIS coding + terminator region by PCR mutagenesis and cloned into pCAMBIA1301 at NcoI and BstEII sites. Primers are listed in Supplementary Table S1. All three constructs were introduced into Col-0 and *tfIIs-1* mutant, by a floral dipping method (53).

### Stress treatments

*Direct heat stress*: for BT, naïve 7-day-old seedlings grown on agar plates were exposed to 45°C in a water bath in the presence of light for 10–30 min. *SAT:* for SAT, seedlings were first acclimated at 37°C for 1 h and then cooled back and kept at 21°C for 1 h. Lethal 45°C heat treatment was applied afterwards in a water bath for 1–2.5 h. *LAT:* for LAT, 5-day-old seedlings were first acclimated at 37°C for 1 h and then cooled back and kept at 21°C for 2 days. Lethal 45°C heat treatment was given afterwards in a water bath for 20–80 min. *TMHT:* for TMHT, 7-day-old seedlings were placed in a growing cabinet preheated to 37°C and kept for 1–5 days. During the treatment, long-day conditions were used (16 h light/8 h dark). Treatments were started at midday (Zeitgeber time, ZT8). *AC*: Gradient acclimation was done in the presence of light in a water bath over 4 h: the temperature rose starting at ZT4 and reached 37°C at ZT7; plants were kept at 37°C for 1 h from ZT7 to ZT8. Plants were cooled back to 21°C following each treatment; samples were taken immediately, or 2 days later at ZT8 for 1d + rec samples. For phenotyping, plants (on agar plates) were cooled back immediately and kept at 21°C for 1 or 2 weeks and then photographed. *Chlamydomonas reinhardtii* (cc-4533) was grown on agar plates (54) and heat stress was done in a water bath. For *Brassica napus* (RV31) and *Hordeum vulgare* (Golden Promise) TFIIS expression analysis, we placed leaf discs (1 cm diameter) of 1-week-old soil-grown plants into hydroponic culture (0.5× MS); AC treatment was done in a water bath as described before (55,56).

### RNA extraction and qRT-PCR

Total RNA was extracted from ∼30 mg seedlings in 600 μl of extraction buffer (0.1 M glycine–NaOH, pH 9.0, 100 mM NaCl, 10 mM EDTA, 2% SDS) and extracted with equal volumes of phenol–chloroform (pH 4.3) and chloroform, precipitated with ethanol and resuspended in sterile water. For qRT-PCR assays, 5 μg total RNA was DNase treated (Ambion AM2222, www.thermofisher.com), precipitated in ethanol and resuspended in sterile water. One microgram of DNase-treated total RNA and random primer was used for the first-strand cDNA reaction (NEB, E6300S, www.neb.com). qPCRs were done using qPCR Master Mix (NEB, M3003S, www.neb.com). qPCR reactions were run in a Light Cycler 96 (Roche) real-time PCR machine. At least three biological samples were assessed in each experiment and standard error bars shown. *P*-values were calculated using unpaired two-tailed Student’s *t*-test to assess the significance of differences; *t*-test comparison of samples is shown where considered relevant. For primers, please see Supplementary Table S1.

### RNA transcriptome and alternative splicing analysis

Total RNA samples obtained from Col-0 and *tfIIs-1* mutant nontreated (NT), 1 h (1h)-, 1 day (1d)- or 1 day + 2 days of recovery (1d + rec)-treated seedlings have been prepared for paired-end Illumina sequencing (in four biological replicates each). Raw RNA-seq data have been made available in the SRA repository (https://www.ncbi.nlm.nih.gov/sra/PRJNA729886). RNA reads were aligned to the *Arabidopsis* genome (TAIR10.v48) obtained from Ensembl Plants (https://plants.ensembl.org/) (57) using hisat2-2.2.1 (58). For differential expression analysis, we used StringTie (v2.1.4) (59) followed by Cuffquant and Cuffdiff (60). Principal component analysis (PCA) plot was created with PCAGO web tool (https://pcago.bioinf.uni-jena.de/), for heatmap Cluster3.0 (de Hoon *et al.*, 2014) and Java Treeview (61). Bedgraph files have been generated using SAMtools 1.12 (62) and visualized by Integrated Genome Browser (v9.1.6) (63). Gene Ontology (GO) analysis was performed with the web tool g:Profiler (https://biit.cs.ut.ee/gprofiler/gost) (64). Alternative splicing (AS) analysis was performed with ASpli (v3.12) (65). Data for U2 and U12 intron identification were acquired from ERISdb database (http://lemur.amu.edu.pl/share/ERISdb/home.html). We visualized intron 5′ and 3′ consensus sequences with WebLogo (https://weblogo.berkeley.edu/) (66).

### Protein extraction and western blotting

Seedlings were homogenized in extraction buffer (150 mM Tris–HCl, pH 7.5, 6 M urea, 2% SDS and 5% μ-mercaptoethanol), boiled for 5 min and cell debris removed by centrifugation at 18 000 × *g* at 4°C for 10 min. The supernatants were resolved on 12% SDS-PAGE, transferred to Hybond PVDF membranes (GE Healthcare) and subjected to western blot analysis. Antibodies used for detection: for 3xHA-tagged HsfA1a, HRP-conjugated antibody (3F10, Roche); for GSy tag (52), anti-GFP antibody (MA5-15256, Thermo Fisher); for sHSP-CI, anti-sHSP17.6 antibody (AS07 254, Agrisera), anti-HSP70 antibody (AS08 371, Agrisera), anti-HSP90-1 antibody (AS08 346, Agrisera), anti-HSP101 (AS07 253, Agrisera), anti-Ub antibody (U5379, Sigma-Aldrich) and anti-SUMO1 antibody (AS08 308, Agrisera); as secondary antibody, we used monoclonal HRP-conjugated anti-mouse (A4416, Sigma-Aldrich) or anti-rabbit (A6154, Sigma-Aldrich). The proteins were visualized by chemiluminescence (ECL kit; GE Healthcare) and quantified by Image Lab 5.1 (Bio-Rad); protein signals have been normalized to Rubisco large subunit (RbcL), having a slow turnover rate (67).

### Protein aggregate purification and detection

Protein aggregate detection was done as described before (49). Briefly, total protein extracts were prepared by homogenization of plant material in 1.0 ml per 0.2 g fresh weight of protein isolation buffer [25 mM HEPES, pH 7.5, 200 mM NaCl, 0.5 mM Na_2_EDTA, 0.1% (v/v) Triton X-100, 5 mM ϵ-amino-*N*-caproic acid, 1 mM benzamidine], using a mortar and pestle and then a Cole-Parmer PTFE glass tissue grinder. The soluble and insoluble fractions were separated from 1.0 ml of total extract by centrifugation at 16 000 × *g* for 15 min at 4°C. The soluble fraction was prepared by adding 0.25 volume of 5× sample buffer and heating for 2 min at 95°C. The insoluble pelleted fraction was washed five times by repeated resuspension in protein extraction buffer containing 0.1 g of quartz sand (Sigma-Aldrich). The pellet (insoluble fraction) was resuspended in 400 ml hot 2× SDS-PAGE sample buffer and clarified by centrifugation at 1500 × *g* for 1 min. Samples were separated by SDS-PAGE and stained; the whole lanes have been quantified by Image Lab 5.1 (Bio-Rad) and ratios calculated.

### Physiological measurements

Photosynthetic activity of 7-day-old seedlings was determined by chlorophyll a fluorescence measurement using a pulse amplitude modulated fluorometer (Imaging-PAM, M-series, Walz, Effeltrich, Germany) completed with a thermoregulatory instrument consisting of a water-cooled Peltier thermoelectric module, a thermocouple thermometer and a control unit. The plants were dark adapted at adequate temperature for 30 min before each measurement, after which the Fv/Fm parameter was determined using a 1.0-s saturated pulse (PPFD = 3000 μmol/m^2^/s) provided by a LED-array illumination unit [IMAG-MAX/L (450 nm)]. Photosynthesis was then activated using 340 μmol/m^2^/s light intensity and the complementary quantum yields, namely the effective quantum yield of photosystem II (PSII) [*Y*(II)], the regulated [*Y*(NPQ)] and the nonregulated [*Y*(NO)], were determined at steady-state conditions according to the nomenclature described earlier (Klughammer and Schreiber, PAM Application Notes 2008 1:27–35. Complementary PSII quantum yields calculated from single fluorescence parameters measured by PAM fluorometry and the saturation pulse method).

### Chromatin immunoprecipitation

For the chromatin immunoprecipitation (ChIP) experiment, we have pre-grown *pTFIIS::GSy-TFIIS;tfIIs-1* seedlings for 6 days (NT samples) and heat treated them for 1 h at 37°C. For each sample, we collected 1 g of raw material and performed ChIP as described before (55). For GSy-TFIIS protein binding, we used the anti-GFP magnetic beads (miltenyibiotech.com, cat. no. 130-091-125). ChIP-qPCR primers are listed in Supplementary Table S1.

## RESULTS

### TFIIS is transcriptionally induced in response to heat

**Figure 1.**
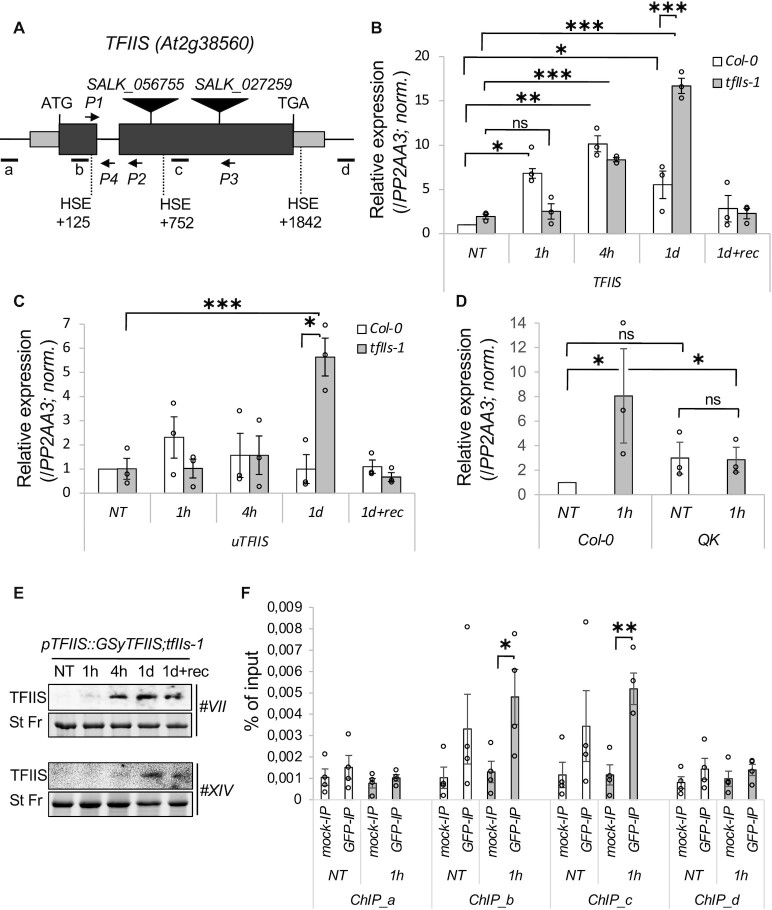
TFIIS is induced by heat. (**A**) Schematic representation of TFIIS gene locus; exons are black boxes and UTR regions are grey boxes; T-DNA insertions are shown above and HSE *cis* elements, genotyping or qRT-PCR (P1, P2, P3, P4) primers are shown below. (**B**) qRT-PCR analysis of TFIIS spliced and (**C**) unspliced mRNA; values were normalized to NT Col-0 plants. (**D**) Relative expression of TFIIS spliced transcripts in *hsfa1a;a1b;a1d;a1e* quadruple knockout (QK) mutant plants. (**E**) Western blot analysis of GSy-TFIIS protein in two independent complementation (*pTFIIS::GSy-TFIIS;tfIIs-1*) lines; stain free as a loading control. (**F**) TFIIS ChIP-qPCR at TFIIS locus; locations of amplicons are shown as horizontal segments in panel (A) (amplicons: a, b, c, d). Bars represent standard errors based on at least three biological replicates; *P*-values based on two-tailed Student’s *t*-test (*P < 0.05, **P < 0.01, ***P < 0.001).

Transcriptional reprogramming is a central pathway of heat adaptation. Our previous RNA-seq analysis (55) showed that the mRNA of the TFIIS accumulates in response to diverse heat treatments (Supplementary Figure S1A) suggesting adaptive roles during HS. To better understand its dynamics, we analysed *TFIIS* mRNA levels during a heat-treated time series in wild-type plants (*A. thaliana* Col-0 ecotype, Figure 1A–C). We measured TFIIS mRNA levels following 1 h (1h), 4 h (4h), 1 day (1d) heat stress at 37°C and 1 day stress followed by 2 days of recovery (1d + rec) treatments. A rapid and dramatic (6–10×) transient accumulation of *TFIIS* mRNA was observed during early heat treatments (1–4 h). Later, the *TFIIS* mRNA levels declined; at 1 day, it was only half of what was measured at 4 h. We also monitored the levels of the unspliced transcript form (*uTFIIS*) as a proxy for transcription activity. The changes in *uTFIIS* level showed a very similar trend to the spliced form (Figure 1C). Thus, we concluded that heat intensifies the transcription of *TFIIS* gene. In accordance with this, we have found three HSE *cis* motifs within the *TFIIS* locus (Figure 1A). Additionally, we re-analysed previously published ChIP-seq data (68) and found that HsfA1b binds to *TFIIS* gene locus. These data suggest that in plants HsfA1 *trans* factors are involved in the transcriptional activation of *TFIIS* (26,51). Indeed, heat did not induce *TFIIS* mRNA accumulation in the *QK* mutant, in which all four members of the HsfA1 TF family are inactivated (Figure 1D) (51).

To prove that heat-triggered transcriptional induction of *TFIIS* is manifested in increased protein amounts, we analysed the level of TFIIS protein in multiple independent *pTFIIS::GSyTFIIS;tfIIs-1* transgenic complementation lines (GSy-tagged TFIIS protein was expressed from the own promoter in a *tfIIs-1* background). As expected, we found that TFIIS protein accumulates significantly in response to heat (Figure 1E).

TFIIS T-DNA insertion mutant (*tfIIs-1*, SALK_056755) has been characterized before (5,6). As the insertion occurs within the coding region (Figure 1A), the TFIIS locus may still be transcriptionally active, and its transcription could be continued and terminated within the T-DNA fragment (generating a chimera *tfIIs-tdna* transcript, referred as such from now on). Thus, we decided to compare the transcriptional activity of the TFIIS gene in the *tfIIs-1* and Col-0 plants by using primers upstream to the insertion point (Figure 1A, P1/P2). Relevantly, the TFIIS transcription was altered in the mutant: at 1 h, *TFIIS* transcript was induced in Col-0 plants, while the *tfIIs-tdna* RNA level was not increased in the *tfIIs-1* mutants; at 4 h, both the *tfIIs-tdna* and *TFIIS* mRNA transcripts accumulated to similar levels; however, under persistent heat (1 day) *TFIIS* mRNA was efficiently suppressed, but the *tfIIs-tdna* RNA in the mutant did not decrease; instead, it was further elevated (Figure 1B). Similar expression dynamics were observed when the accumulation of unspliced transcript forms was analysed (P1/P4 primers); the unspliced *uTFIIS* transcript was rapidly and transiently induced, while the unspliced *utfIIs-tdna* RNA accumulation trailed behind and was further elevated at 1 day heat treatment (Figure 1C). Notably, we did not detect any transcript when we used primers that produced amplicon spanning the T-DNA insertion point (Supplementary Figure S1C and D, P1/P3 primers).

The observations that in the mutant heat-induced transcriptional activation is slow and that transcription is not repressed at persistent heat suggest that TFIIS protein itself could play a role in the transcriptional regulation of the TFIIS locus. To analyse whether the TFIIS locus is autoregulated, we performed a ChIP experiment using the TFIIS complementation line. The TFIIS protein associated with the transcribed region but did not associate with exogenic upstream or downstream regions (Figure 1F). TFIIS protein binding to the TFIIS gene was mildly heat induced. These data raise the possibility that TFIIS potentially contributes to its own expression.

### TFIIS heat induction is widely conserved in plant kingdom

Heat-induced upregulation of TFIIS transcription suggests a functional requirement during heat adaptation. To back this hypothesis, we tested the conservation of TFIIS’s heat regulation. First, we searched for the putative TFIIS homologues in unicellular alga *C. reinhardtii*, dicot *B. napus* and monocot *H. vulgare*. The alignments were performed using Clustal Omega multiple protein sequence alignment (https://www.ebi.ac.uk/Tools/msa/clustalo/) and sequences were derived from Ensembl Plants (https://plants.ensembl.org/). We have identified a single TFIIS-like protein-coding gene in *C. reinhardtii* (CreTFIIS), three genes in *B. napus* (BnaTFIISa, b and c) and one gene in *H. vulgare* (HvTFIIS) based on their similarity and identity to *A. thaliana* homologue gene (Supplementary Figures S2 and S3). All these putative TFIIS proteins encoded by CreTFIIS, BnaTFIIS and HvTFIIS genes have a significant similarity and identity to *Arabidopsis* homologue (Supplementary Figures S2 and S3), harbour the three characteristic domains, are predicted to form the four-helix bundle within domain I and the three-helix bundle in domain II (CreTFIIS domains I and II are less conserved), and contain the zinc ribbon motif with the four zinc-chelating cysteine residues and the invariant DE dipeptide within domain III (5,11,13). Based on their domain organization, predicted structures and presence of catalytic core, these genes likely encode *bona fide* TFIIS factors.

**Figure 2.**
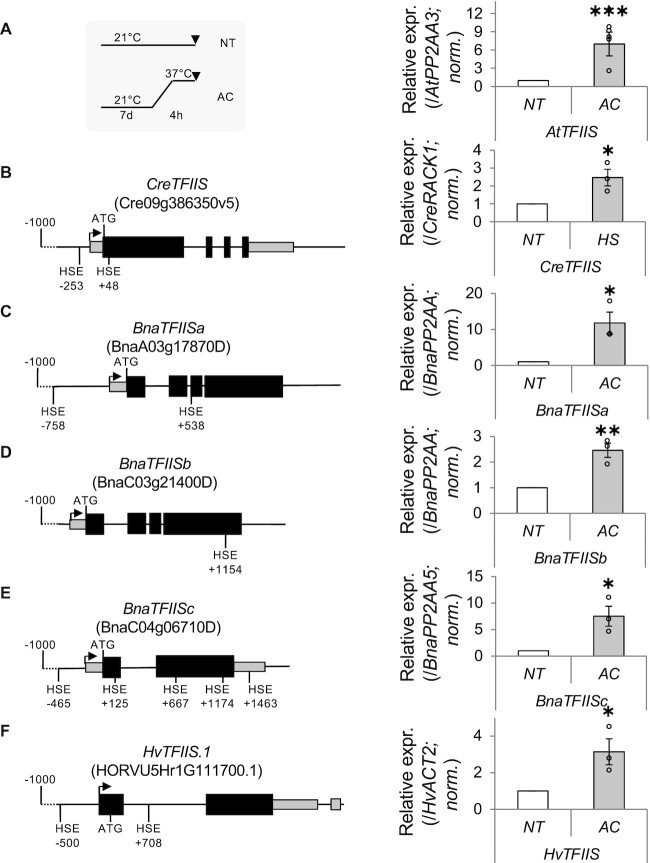
Heat induction of TFIIS is conserved among distant plant species. (**A**) Schematic representation of the heat acclimation (AC) programme used (left panel) and induction of TFIIS in *A. thaliana* (right panel); (left panels) schematic representation of TFIIS homologous genes in *C. reinhardtii* (**B**), *B. napus* (**C**–**E**) and *H. vulgare* (**F**); exons are shown as black boxes, introns are shown as lines, UTRs are shown as grey boxes, ATG start codons are shown above and HSE *cis* elements are shown below. qRT-PCR of TFIIS gene homologous is shown (**A**–**F**, right panels). Bars represent standard errors based on at least three biological replicates; *P*-values based on two-tailed Student’s *t*-test (*P < 0.05, **P < 0.01, ***P < 0.001).

To test whether these genes are expressed and heat responsive, we analysed their mRNA changes during ambient and heat acclimation conditions. We have found that *CreTFIIS*, *BnaTFIISa*, *b* and *c*, and *HvTFIIS* mRNAs were all expressed, and significantly induced in response to heat (Figure 2B–F). Additionally, we detected HSE *cis* elements within all these loci, suggesting that HSFs may be involved in their heat induction. The widely conserved regulation of TFIIS in several distant plant species (including a unicellular life form, dicot and monocot crops) having markedly different lifestyles (aquatic versus terrestrial) and separated by ∼500 Mya evolution suggests functional relevance for TFIIS heat induction.

### TFIIS is necessary for heat tolerance

**Figure 3.**
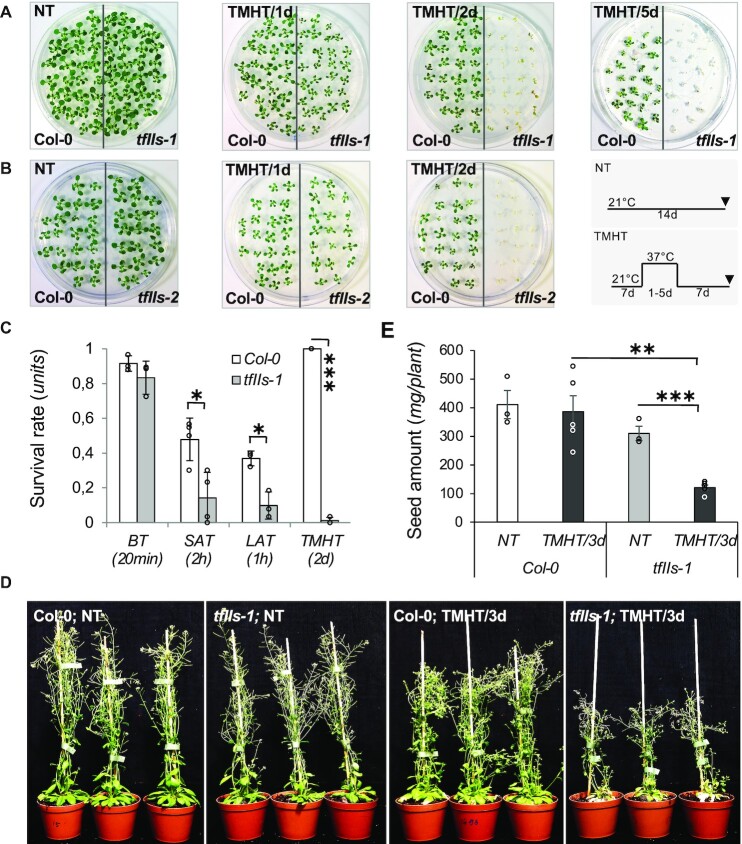
TFIIS is essential for heat stress adaptation throughout the life cycle of *A. thaliana*. TMHT of (**A**) *tfIIs-1* or (**B**) *tfIIs-2* mutant plants compared to Col-0; schematic representation of TMHT regime is shown on the right. (**C**) Survival rate of Col-0 and *tfIIs-1* plants to BT, SAT, LAT and TMHT regimes. (**D**) Soil-grown flowering Col-0 and *tfIIs-1* mutant plants and (**E**) seed amounts collected from these following TMHT treatment lasting 3 days (TMHT/3d). Bars represent standard errors based on at least three biological replicates; *P*-values based on two-tailed Student’s *t*-test (*P < 0.05, **P < 0.01, ***P < 0.001).

To justify the biological role of TFIIS induction in HSR, we analysed the heat sensitivity of the previously characterized *tfIIs-1* (SALK_056755) and *tfIIs-2* (SALK_027259) T-DNA lines (5,6). For this, we employed various heat treatments, including BT, SAT, LAT and TMHT programmes. The growth of *tfIIs-1* seedlings exposed to BT was somewhat retarded in comparison to Col-0 plants, suggesting a minor requirement of TFIIS (Supplementary Figure S4). Significant differences in survival rates were found between *tfIIs-1* mutant and Col-0 plants in response to SAT, LAT and TMHT regimes (Figure 3 and Supplementary Figure S4). The most striking difference was observed in response to TMHT: the growth of the *tfIIs-1* mutant seedlings was retarded following 1 day 37°C heat treatment, and all seedlings died following 2 days of TMHT treatment, while Col-0 plants were able to survive even 5–7 days at 37°C (Figure 3A). We got similar results when we tested thermotolerance of *tfIIs-2* mutant (Figure 3B). In summary, the sublethal 37°C heat (to Col-0) becomes lethal upon TFIIS absence.

Complementation assays were conducted to prove that the lack of TFIIS protein is responsible for the heat-sensitive phenotype of *tfIIs-1*. Two different types of complementation lines were subjected to TMHT: *pTFIIS::GSyTFIIS;tfIIs-1* and *p35S::MycTFIIS;tfIIs-1* (in the *p35S::MycTFIIS;tfIIs-1* plants, the Myc-tagged TFIIS protein was expressed from the constitutive 35S promoter). Relevantly, the heat-sensitive phenotype of *tfIIs-1* was reverted by both constructs in several independent lines (Supplementary Figure S5A and B). We have also tested thermotolerance of overexpressor lines, including *p35S::GSyTFIIS;tfIIs-1*, *p35S::GSyTFIIS;Col-0* or *p35S::MycTFIIS;Col-0*, employing different TMHT and LAT regimes, but could not observe significant improvement in survival or growth rate compared to wild-type seedlings.

To clarify whether TFIIS is required for heat tolerance only at seedling stage or it is also important at other developmental stages, we investigated the heat sensitivity of *tfIIs-1* mutant during flowering (Figure 3D). Col-0 control plants were mildly affected by 3 days of TMHT treatment: leaf yellowing, petal wilting and pollination defects were observed 1 week after stress. After a couple of weeks, however, the plants resumed their growth and fertilization, producing normal amount of seeds. The *tfIIs-1* plants were more seriously affected by the treatment: almost all rosette leaves and siliques got yellow and died; the central growing floral stem died back; regrowth was resumed very late from secondary, auxiliary buds; and *tfIIs-1* plants remained small and produced shorter siliques with few seeds (Figure 3D and E). These results show that TFIIS is needed for heat adaptation throughout the whole life cycle of plants to support both survival and reproduction success.

The observations that TFIIS is expressed throughout all plant body and is required during diverse HS regimes (Figure 3 and Supplementary Figures S4 and S5) raised the possibility that it functions as a general protective factor during abiotic stress conditions. To pursue this possibility, first we analysed TFIIS changes in the *Arabidopsis* eFP Browser database (https://bar.utoronto.ca/efp/cgi-bin/efpWeb.cgi). Apart from heat induction, the expression of TFIIS is not markedly altered by other abiotic stresses, including cold, salt, drought, osmotic, genotoxic stress or wounding. Next, we experimentally tested salt tolerance of *tfIIs-1* mutants compared to Col-0: no differences in growth and development between the two genotypes could be found in response to different salt concentrations, except a marginal difference at 160 mM salt (Supplementary Figure S6A and B). Based on these, we excluded TFIIS of being a universal anti-stress factor. Whether TFIIS has roles under other types of stress responses needs further investigations.

### TFIIS enables efficient transcriptome reprogramming during HSR

**Figure 4.**
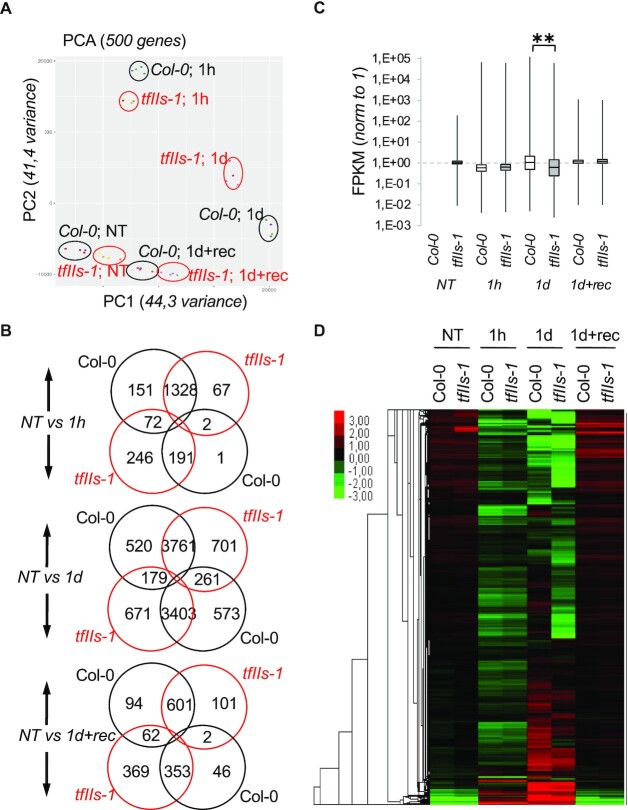
Transcriptome analysis of heat-induced changes in Col-0 and *tfIIs-1* plants. (**A**) PCA of transcriptome samples of Col-0 or *tfIIs-1* plants. (**B**) Differentially up- or downregulated transcript numbers (minimum 2-fold change, *P* < 0.01), NT compared to 1 h (1h) and 1 day (1d) heat stress, or 1 day heat treatment followed by 2 days of recovery (1d + rec) treatments (upper, middle and lower panels, respectively). (**C**) Normalized FPKM changes and (**D**) heatmap analysis of significantly changed transcripts without fold-change restrictions during a heat-treated time series in Col-0 and *tfIIs-1* plants; heatmap colour legend is shown on the left; *P*-values based on two-tailed Student’s *t*-test (**P < 0.01).

As TFIIS is a general transcription factor, we reasoned that it regulates thermal adaptation at the transcriptional level. To explore genome-wide transcriptional differences between Col-0 and *tfIIs-1* plants, we performed a whole transcriptome analysis (Supplementary Table S2). Samples were collected from NT, 1 h and 1 day heat-stressed and recovery (1d + rec) plants. Based on the PCA, the biological replicates correlated very well with each other (Figure 4A). Reads mapping to TFIIS gene locus were checked and the presence and place of T-DNA insertion in *tfIIs-1* plants was confirmed.

We filtered the data based on statistically significant (*P* < 0.01) differential expression of at least 2-fold between Col-0 and *tfIIs-1* (Supplementary Table S1). At NT conditions, the differences between the two genotypes were minor, consistent with the phenotype and earlier findings (5). After 1 h heat, 1551 and 1397 transcripts were upregulated in Col-0 and *tfIIs-1* plants, respectively (Figure 4B, top diagram); 194 and 509 transcripts were specifically downregulated in Col-0 and *tfIIs-1*, respectively. The predominant part of upregulated transcripts (1328) was common in Col-0 and *tfIIs-1* representing 85.6% and 95% of totals. GO analysis (GO terms, Supplementary Figure S7) of commonly upregulated transcripts revealed enrichment of transcripts involved in HSR, protein folding and hypoxia, which is consistent with the treatment. The high overlap between genotypes in the upregulated RNA species may suggest that TFIIS is not required for the induction of these mRNAs; however, quantitative differences between Col-0 and mutant may still exist (see later). Interestingly, only 3 transcripts (1.5%) were specifically downregulated in Col-0, but we have detected 318 transcripts (62%) being specifically downregulated in *tfIIs-1* plants. This is consistent with the positive role of TFIIS in the transcription process and suggests that TFIIS is required to maintain efficient transcription of these mRNAs during HSR; GO term analysis revealed that the specifically downregulated transcripts belong to groups such as cell wall organization, phenylpropanoid biosynthesis, antioxidant activities, Casparian strip and suberin biosynthetic processes, suggesting alterations of cell wall structure specifically in the mutant plants. Interestingly, transcripts responding to oxidative stress and having oxidoreductase activities were also among the transcripts commonly upregulated in Col-0 and downregulated in *tfIIs-1* (72, representing 4.6% and 14%, respectively). Therefore, it seems that oxidative stress pathways are rather induced in Col-0 but potentially suppressed in *tfIIs-1* plants.

Much bigger differences were observed when samples were compared following 1 day heat treatment, including difference to NT and difference between genotypes (Figure 4B, middle diagram). On average, about the same number of transcripts were up- and downregulated compared to NT samples: 4460 transcripts accumulated in Col-0 (with 699 only in Col-0, 15.6%), while 4723 in the mutant (962 only in *tfIIs-1*, 20%); 4237 transcripts were downregulated in Col-0 (837 only in Col-0, 19.6%), while 4253 were downregulated in the mutant (850 only in *tfIIs-1*, 19.9%). The high number of transcript changes in both genotypes suggests secondary and indirect effects caused by the persistent heat exposure. The GO terms of commonly upregulated transcripts (3761, representing 84.3% and 79.6% in Col-0 and mutant, respectively) were transcripts involved in RNA binding or processing, ribosome biogenesis and translation, while the commonly downregulated transcripts (3403, 80.3% and 80%) were involved in response to abiotic stimulus and diverse metabolic processes (Supplementary Figure S8). We analysed the GO terms of specifically up- and downregulated transcripts as well. The Col-0-specific upregulated transcripts were involved in nucleic acid metabolism, nitrogen compounds and methyltransferase activities, while *tfIIs-1*-specific upregulated transcripts were involved in protein repair, abiotic stimulus, light responses, chloroplast and endomembrane system components. The GO terms of downregulated transcripts specific for Col-0 were chloroplast components and photosynthesis transcripts, while in *tfIIs-1* these were tissue development, actin cytoskeleton and Golgi membrane components. Of note, the same RNA metabolism factors were upregulated in Col-0 and downregulated in *tfIIs-1*, while common chloroplast and photosynthesis-linked factors were downregulated in Col-0 and some upregulated in *tfIIs-1*.

Finally, we analysed transcriptome changes of recovered plants (NT versus 1d + rec). Based on PCA and heatmap analysis, the transcriptome differences between treatments and genotypes were markedly reduced (Figure 4A and B). Seven hundred fifty-seven transcripts were found to be upregulated in Col-0 with 704 upregulated in the *tfIIs-1* mutant (Figure 4B, bottom diagram). Of these, most transcripts (601, 79.4% and 85.3%, respectively) were commonly heat responsive in both genotypes. The downregulated transcript pools, however, were slightly different: out of 401 transcripts that were downregulated transcripts in Col-0, only 48 (11%) were Col-0-specific, but out of total 784 downregulated transcripts, 431 (55%) were specifically altered in the mutant only. Col-0-specific upregulated transcripts were enriched for genes involved in response to stimulus, biotic stress, glutathione metabolism and detoxification GO terms, while *tfIIs-1*-specific transcripts were enriched in the GO terms chemical stimulus and hormonal pathways (Supplementary Figure S9). Col-0-specific downregulated transcripts were categorized to auxin-hormonal pathway and response to chemical stimulus GO terms, while *tfIIs-1*-specific downregulated transcripts were enriched primarily in cell wall organization and biosynthesis GO terms. Of note, *tfIIs-1* plants do not die following a day of TMHT treatment but were able to efficiently recover although with a retarded growth capacity (Figure 3A–C and Supplementary Figure S4D).

To further reveal the difference between the two genotypes, we directly compared the transcriptomes of the *tfIIs-1* mutant with Col-0 at each condition and conducted GO enrichment analysis on differentially expressed genes (Supplementary Figure S10). This comparison highly recapitulated our previous observations (Supplementary Figures S7–S10). Notably, transcripts belonging to GO terms like unfolded protein binding, heat shock, and response to stress and temperature were enriched in Col-0 at 1 h (Supplementary Figure S10B), suggesting quantitative difference between these transcripts in the two genotypes. These data strongly support the notion that early HS response is dramatically impaired in the *tfIIs-1* mutant.

In summary, although a large number of up/downregulated transcript differences between the Col-0 and the *tfIIs-1* mutant are likely the indirect consequences of the TFIIS absence (especially at 1 day), a clear separation of transcriptome profile emerges upon persistent heat treatment, with Col-0 transcriptome leaning to reshape the RNA metabolism, efficiently downregulate photosystem-linked transcripts and hormonal pathways, but switching on detoxification; on the contrary, *tfIIs-1* persists producing photosynthesis-linked transcripts and hormonal responsive transcripts, but has decreased levels of transcripts required for oxidative stress response and structural components like epidermis development, cytoskeleton and cell wall. Col-0 plants switch efficiently to a stress management transcriptome programme, while *tfIIs-1* plants lag in this shift.

To get a wider view on the transcriptome dynamics, we have further analysed the transcriptome differences in response to heat between the two genotypes by including all significantly changing transcripts (*P* < 0.01) without fold-change restrictions (Supplementary 4C and D). During early (1 h) heat treatment, a marked but similar downregulation of overall transcriptome was observed in both genotypes. Upon persistent heat (1 day), however, this trend was markedly changed and a clear separation of the two genotypes could be observed: in the Col-0 transcriptome, significantly more transcripts were expressed at higher level compared to *tfIIs-1* mutant (Figure 4C). Of note, majority of these transcripts have small fold-change differences. The downward shift of the *tfIIs-1* mutant transcriptome was also clearly visible on the heatmap chart (Figure 4D).

In conclusion, the absence of TFIIS directly or indirectly impacts a large number of transcripts (*n* = 14 553) significantly, although mildly. These data suggest that TFIIS activity is not required for regulation of selected/specific cascades of heat adaptation pathways but aids a prompt transcriptome shift to stress response programme. However, it cannot be excluded that TFIIS is required to efficiently express a few master regulators that are required for this transcriptome reprogramming.

### TFIIS is required for proper AS during heat stress

It was proposed that AS is an essential component of heat stress response and thermo-memory (69). Based on the ‘kinetic coupling’ model, transcriptional elongation rates modulate AS; fast elongation reduces the splicing frequency of weak promoter-proximal splice sites, while slow elongation has the opposite effect (70,71). TFIIS promotes transcription elongation rate; thus, it can indirectly modulate AS (15,16).

**Figure 5.**
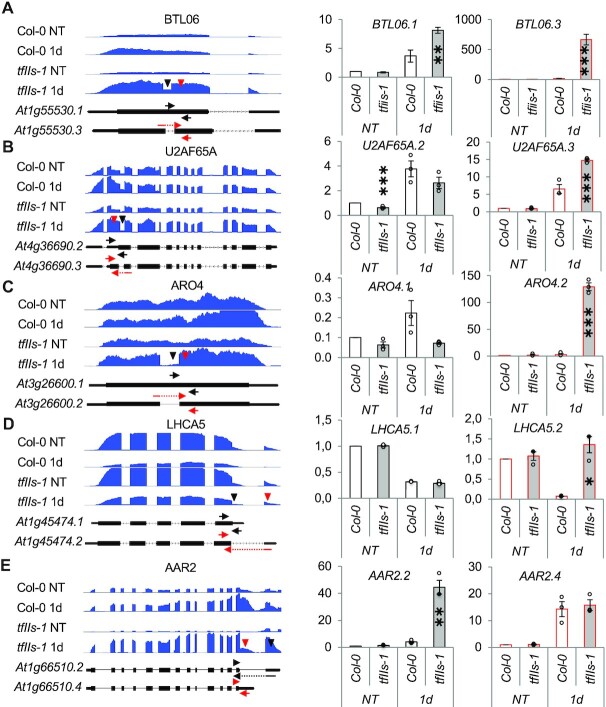
AS event changes during heat treatment in Col-0 and *tfIIs-1*. Genome browser images of transcriptome read tracks on selected loci with altered AS events (left panel; black and red triangles point to the two AS events); splicing isoform ratios were quantified by qRT-PCR (right panels; *Y*-axis represents relative expression norm to *PP2AA3*). Gene names, genotypes and schematic representation of splicing variants alongside the relevant AS primers (black and red, respectively) are shown; bars represent standard errors based on three biological replicates; differences between genotypes at each conditions were compared; *P*-values based on two-tailed Student’s *t*-test (*P < 0.05, **P < 0.01, ***P < 0.001).

To better understand the impact of TFIIS on splicing during heat stress conditions, we analysed AS changes in our transcriptome using the ASpli *in silico* tool (https://bioconductor.org/packages/release/bioc/html/ASpli.html) (Figure 5, Supplementary Figure S11 and Supplementary Table S3). Although heat had a significant impact on AS events, we focused our study on the AS differences between the two genotypes. In accordance with previous findings, under NT conditions the AS pattern was similar in Col-0 and *tfIIs-1* plants; only six AS transcripts were specifically affected in the mutant background (15).

In contrast, the AS patterns of heat-stressed plants were significantly different; we identified 86 and 1760 AS events that differed between the Col-0 and *tfIIs-1* plants at 1 h and 1 day heat treatment, respectively. Recovery period of 2 days resulted in efficient reversal of splicing defect, as only six AS events were found to be altered in *tfIIs-1* plants compared to Col-0. Thus, TFIIS is required for the proper AS under heat stress conditions.

The predominant part of AS differences observed between Col-0 and *tfIIs-1* plants was categorized by ASpli as intron retentions (IRs): during 1 h and 1 day treatment, 62% and 59% of AS events were classified as IRs, respectively (Figure 5, Supplementary Figure S11 and Supplementary Table S3). IR splicing differences occurred in both ‘directions’; they were either upregulated in Col-0 plants (introns being skipped in Col-0 and/or more efficiently spliced in *tfIIs-1* plants) or upregulated in *tfIIs-1*. Remarkably, 76% of introns at differential IR sites were more efficiently spliced out in *tfIIs-1* at 1 day, as predicted by the kinetic coupling model. Several of these appeared only in the heat-treated *tfIIs-1* mutant sample but were not detected in Col-0 transcriptome at any extent, suggesting the usage of novel cryptic splice sites within these transcripts (Supplementary Figure S11C–I, M, Q and T).

We further studied IR events/cryptic splicing in detail: of these, 211 events occurred in exons and 574 occurred within introns. Average lengths of differentially spliced introns were 156 bp (*Arabidopsis* average is 173 bp) and average exon size was 151 bp (*Arabidopsis* average is 172 bp); these introns are canonical U2 type (Supplementary Figure S12) as only 2.7% (21/786) were annotated as U12-type introns. Intron splicing alterations occurred mostly in longer genes, with an average gene length of 3.9 kb (gene length average in *A. thaliana* is 2.4 kb); the average distance of altered splicing events was 1937 bp from the transcription start site (TSS) and 837 bp from the termination site. Some transcripts were alternatively spliced at multiple intron sites (Supplementary Figure S11B, J and T). In 251 cases (24%), we observed that alternative introns were retained in the *tfIIs-1* background (Supplementary Figure S11T and Supplementary Table S3). Besides IR, absence of TFIIS altered other types of AS events (for details see Supplementary Table S3). In a few cases, the absence of TFIIS altered transcription initiation (Supplementary Figure S11J) or termination site selection during persistent heat treatment (Figure 5D and E, and Supplementary Figure S11N).

To validate the AS changes detected in the RNA transcriptome analysis with an alternative method, we conducted qRT-PCR measurements on several transcript AS pair representatives for IR/cryptic intron splicing (At1g55530, At4g36690, At3g26600) or alternative termination (At1g45474, At1g66510) (Figure 5A–E). For this, we focused the analysis on NT versus 1 day treatment, where the differences were the most obvious. Heat stress induction and differential changes of the AS isoforms due to the *tfIIs-1* background were confirmed in every case. To analyse whether the AS at these loci is a consequence of TFIIS absence, we conducted TFIIS ChIP-qPCR. TFIIS was mildly enriched within the examined loci (Supplementary Figure S13). TFIIS binding was not significantly affected by heat treatment at most of the analysed genes, or was even reduced at LHCA5, in accordance with transcriptional downregulation of this locus (see later).

We also analysed the GO terms of differentially spliced genes and have found that they were enriched for heat stress, response to stimuli and protein unfolding at 1 h, and RNA binding, photosynthesis and metabolic pathway at 1 day (Supplementary Table S4). Notably, these terms were enriched also when we studied DEGs ‘in general’.

To unravel a potential link between gene expression level and *tfIIs-1*-dependent splicing patterns, we specifically analysed the expression of AS genes (Supplementary Figure S14 and Supplementary Table S3). We observed that overall AS gene transcripts show a lower abundance in *tfIIs-1* compared to Col-0 at 1 h heat treatment (Supplementary Figure S14A). Regarding AS subgroups, genes producing *tfIIs-1*-specific intron containing transcripts (being more strongly spliced in *tfIIs-1*) tended to have higher expression in *tfIIs-1*. At 1 day, the overall gene expression was higher in Col-0 compared to *tfIIs-1* (Figure 4C and Supplementary Figure S14B) and AS genes had similar behaviour; the subgroups of genotype-specific intron splicing were correlated with a shift towards higher expression (Supplementary Figure S14B).

In conclusion, the absence of TFIIS alters the AS pattern of hundreds of genes/transcripts under heat stress conditions.

### TFIIS is required for accurate HSP expression

Transcription of HSPs is key element of heat response. As we found that TFIIS plays a critical role in transcriptional reprogramming during heat treatment (Figure 4 and Supplementary Figures S7–S10), we hypothesized that it is involved in the regulation of HSP expression. Indeed, when we analysed HSP family transcripts in our RNA transcriptome dataset, we observed that most components of sHSP (15–27 kDa), HSP60, HSP70 and HSP90 chaperone families were induced at significantly lower level in the mutant *tfIIs-1* compared to wild-type plants at 1 h or 1 day (early and late transcripts, respectively), and that the expression of several *HSP* mRNAs was not suppressed to Col-0 levels at 1 day (Supplementary Figure S15). Notably, mostly the level of *sHSPs* was maintained or even further induced at 1 day.

**Figure 6.**
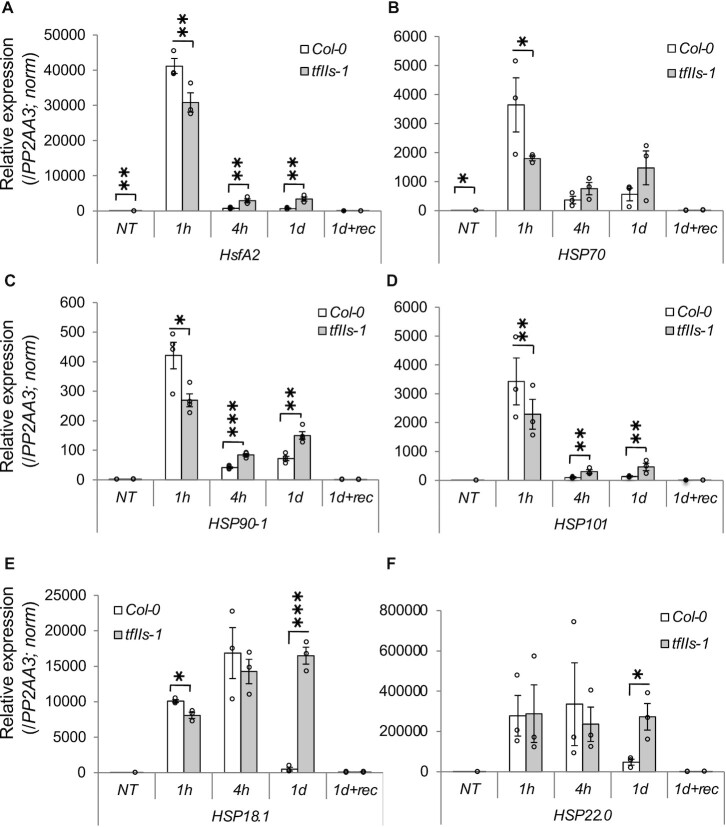
The expression of heat stress response transcripts is altered in the absence of TFIIS. Relative expressions of (**A**) *HsfA2*, (**B**) *HSP101*, (**C**) *HSP70*, (**D**) *HSP90-1*, (**E**) *sHSP18.1* and (**F**) *sHSP22.0* transcripts as measured by qRT-PCR analysis; treatment conditions as in Figure 4; bars represent standard errors based on at least three biological replicates; *P*-values based on two-tailed Student’s *t*-test (*P < 0.05, **P < 0.01, ***P < 0.001).

To validate the transcriptome changes, we analysed the expression of multiple players of the HSR pathway by qRT-PCR. For this, we measured the transcript levels of *HsfA2*, *HSP70*, *HSP90*, *HSP101*, *HSP18.2* and *HSP22.0* as representative factors involved in different aspects of HSR (51,72–75) (Figure 6). Significant differences were observed already at NT in the case of *HsfA2* and *HSP70* transcripts; all assayed transcripts were induced at the early phase of HSR (1 h), but accumulation of *HsfA2*, *HSP70*, *HSP90-1*, *HSP101* and *sHSP18.1* transcripts was significantly lower in *tfIIs-1* mutant plants. At 4 h or 1 day, the transcription attenuation of these loci was inefficient in *tfIIs-1* mutants (Figure 6). The most striking difference at the 1 day time point was detected in the case of sHSP transcripts, which were not repressed but further maintained (Figure 6E and F). Following the recovery period (1d + rec), the levels of all transcripts returned to basal level in both Col-0 and *tfIIs-1* plants.

**Figure 7.**
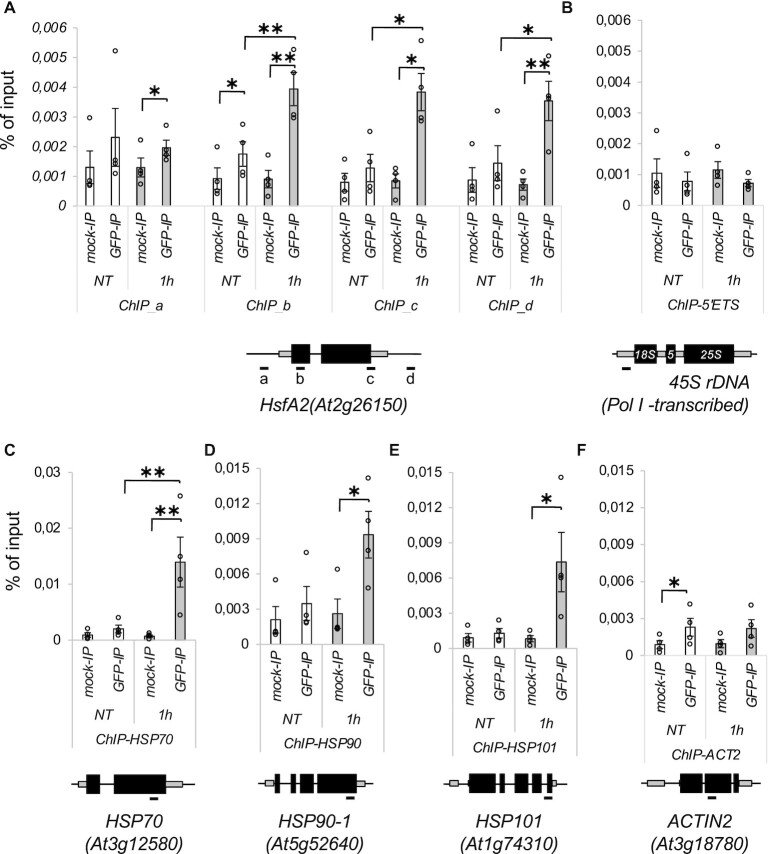
TFIIS is associated with Pol II-transcribed gene loci in a heat-dependent manner. ChIP-qPCR analysis of GSy-TFIIS binding to (**A**) HsfA2, (**B**) 45S ribosomal locus, (**C**) HSP70, (**D**) HSP90, (**E**) HSP101 and (**F**) ACT2 gene loci. 45S rDNA locus is transcribed by Pol I; therefore, it was used as a negative control. Locations of qPCR amplicons are shown as horizontal segments below the schematic of each locus (not to scale); bars represent standard errors based on at least three biological replicates; *P*-values based on two-tailed Student’s *t*-test (*P < 0.05, **P < 0.01, ***P < 0.001).

To directly show the requirement of TFIIS for the early transcriptional activation of HS transcripts, we measured TFIIS binding to these loci by ChIP-qPCR. The TFIIS protein bound within the gene body of these Pol II-transcribed genes (Figure 7A and C–E) but was absent at Pol I-transcribed 45S ribosomal loci (Figure 7B). Importantly, TFIIS localization was consistently enhanced upon heat stress at Pol II HS-induced genes but not at housekeeping *Actin2* gene (Figure 7F).

**Figure 8.**
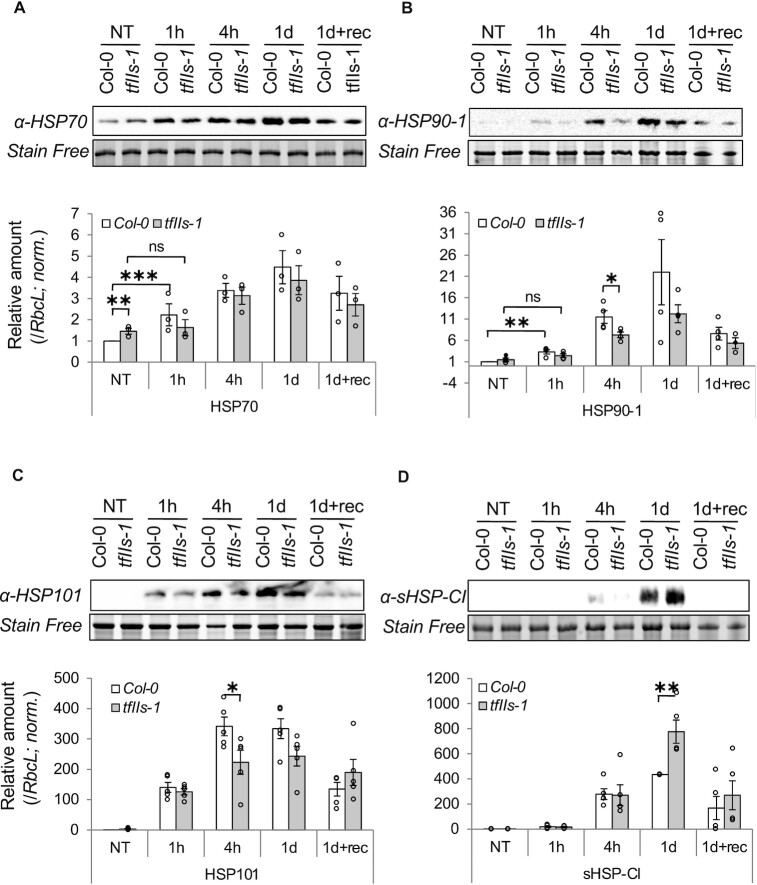
HSP protein accumulation is affected in *tfIIs-1* mutant in response to heat. Western blot analysis of (**A**) HSP70, (**B**) HSP90-1, (**C**) HSP101 and (**D**) sHSP-CI proteins (left); stain-free images of RbcL are shown as loading controls. Quantifications of at least three experiments are shown on the right; treatment conditions as in Figure 4; bars represent standard errors based on at least three biological replicates; *P*-values based on two-tailed Student’s *t*-test (*P < 0.05, **P < 0.01, ***P < 0.001).

To verify that the delayed transcriptional induction of HS transcripts in the absence of TFIIS results in differences at protein level, we compared HSP protein amounts during heat in Col-0 and *tfIIs-1* plants. Consistent with the mRNA levels, we have found that accumulation of HSP70 and HSP90 proteins was significantly delayed in the mutant at early timepoints (1–4 h, Figure 8A and B). At later stages, considerable differences could be observed in the amount of HSP90 and HSP101 chaperons (4 h to 1 day; Figure 8B and C). Notably, the ATP-independent sHSP-CI chaperones accumulated to a markedly higher level in the *tfIIs-1* mutant at 1 day heat treatment (Figure 8D). The persistent transcription of HSPs (Figure 6 and Supplementary Figure S15) and the enhanced protein accumulation of sHSPs in the late heat stress period (Figure 8D) suggest elevated level of proteotoxic stress in the *tfIIs-1* mutant plants.

### Absence of TFIIS indirectly induces proteotoxic stress

**Figure 9.**
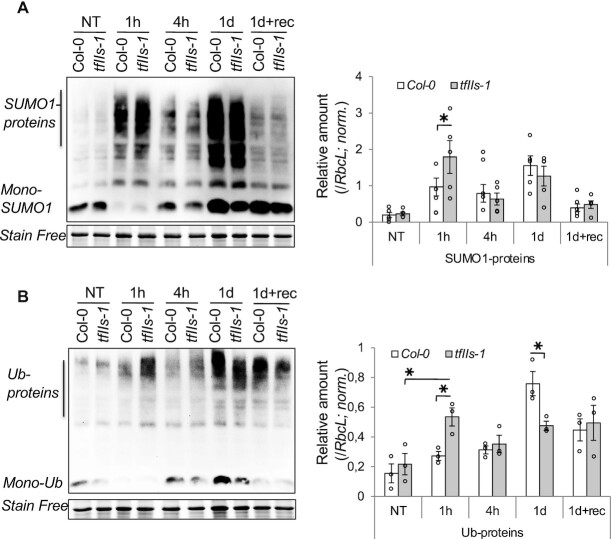
Post-translational modification of proteome is altered in the absence of TFIIS. Sumoylation (**A**) and ubiquitination (**B**) post-translational modifications were measured by western blot analysis in Col-0 and *tfIIs-1* plants; stain-free images of RbcL are shown as loading controls (left); treatment conditions as in Figure 4. Quantifications are shown on the right; bars represent standard errors based on at least three biological replicates; *P*-values based on two-tailed Student’s *t*-test (*P < 0.05, **P < 0.01, ***P < 0.001).

Moderately high temperature stress results in increased thermosensitive protein denaturation and subsequent post-translational modification, like sumoylation and ubiquitination. Although both modifications can alter the functionality of target proteins in many ways, sumoylation (in general) transiently impedes protein aggregation and enables ubiquitination, while ubiquitinated proteins are being either refolded by the help of HSP chaperones or degraded by the proteasome (49,76–78). We examined global changes in the amount of sumoylated and ubiquitinated proteins in Col-0 and *tfIIs-1* plants. Although at 1 h the accumulation of sumoylated and ubiquitinated conjugates was observed in both genotypes, in the *tfIIs-1* mutant significantly higher levels of these conjugates were measured (Figure 9A and B). After 4 h heat, both sumoylated and ubiquitinated protein amounts were decreased, probably due to the combined activity of HSP-mediated recycling of these conjugates and enhanced proteasome activity, as shown earlier (78). Oppositely, 1 day heat treatment induced significantly higher level of ubiquitinated proteins in Col-0 plants (Figure 9B). Notably, we observed a consistent shift of ubiquitin conjugates’ signal towards higher molecular weight range during and following persistent heat treatment in Col-0 plants, suggesting the presence of larger polyubiquitinated side chains on proteins. The amount of sumoylated and ubiquitinated proteins declined in both Col-0 and mutant plants during the recovery period (Figure 9A and B). These alterations confirm altered proteostasis in the *tfIIs-1* plants, consistent with the delayed induction of chaperones and aberrantly spliced transcripts.

**Figure 10.**
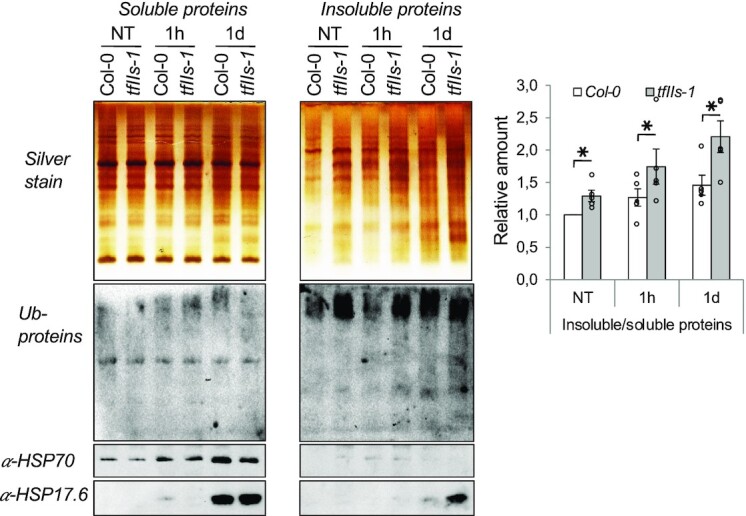
Insoluble protein aggregates accumulate to higher level in *tfIIs-1* mutant plants in response to heat. Silver staining gel images of soluble and insoluble protein fractions from Col-0 and *tfIIs-1* plants are shown (top left); sister gels were analysed for ubiquitin conjugates, HSP70 and sHSP-CI contents (bottom left); treatment conditions as in Figure 4. Quantifications of silver-stained soluble and insoluble protein amount ratios are shown (right); bars represent standard errors based on at least three biological replicates; *P*-values based on two-tailed Student’s *t*-test (*P < 0.05).

To directly test the extent of proteotoxic stress in Col-0 and *tfIIs-1* plants, we purified and compared the amounts of insoluble proteins at NT, 1 h and 1 day heat-treated time points between the genotypes. A significant increase was observed in the amount of protein aggregates in response to heat, as shown earlier (47,49). Importantly, in the *tfIIs-1* mutant plants the amount of insoluble proteins was significantly increased under NT conditions and in response to heat (Figure 10). We also confirmed that the majority of ubiquitinated conjugates are present in the insoluble fractions along with sHSPs, but not HSP70, as shown before (47,49).

### Photosynthetic activity is impaired in *tfIIs-1* under heat stress conditions

Inhibition of photosynthesis is reversible when temperature is only moderately higher than the optimal for a relatively short period, but permanent damage will occur if heat stress is persistent and photosynthetic proteins are difficult to replace (79,80). Accumulation of HSPs is important to provide protection for various photosynthetic apparatus components (81,82). Impaired proteostasis in *tfIIs-1* plants will lead to collapse of photosystem apparatus and activity. Altered accumulation (GO terms) of transcripts involved in photosynthesis and chloroplast development following long-term heat treatment also suggested marked differences in photosynthetic activities between Col-0 and mutant plants.

**Figure 11.**
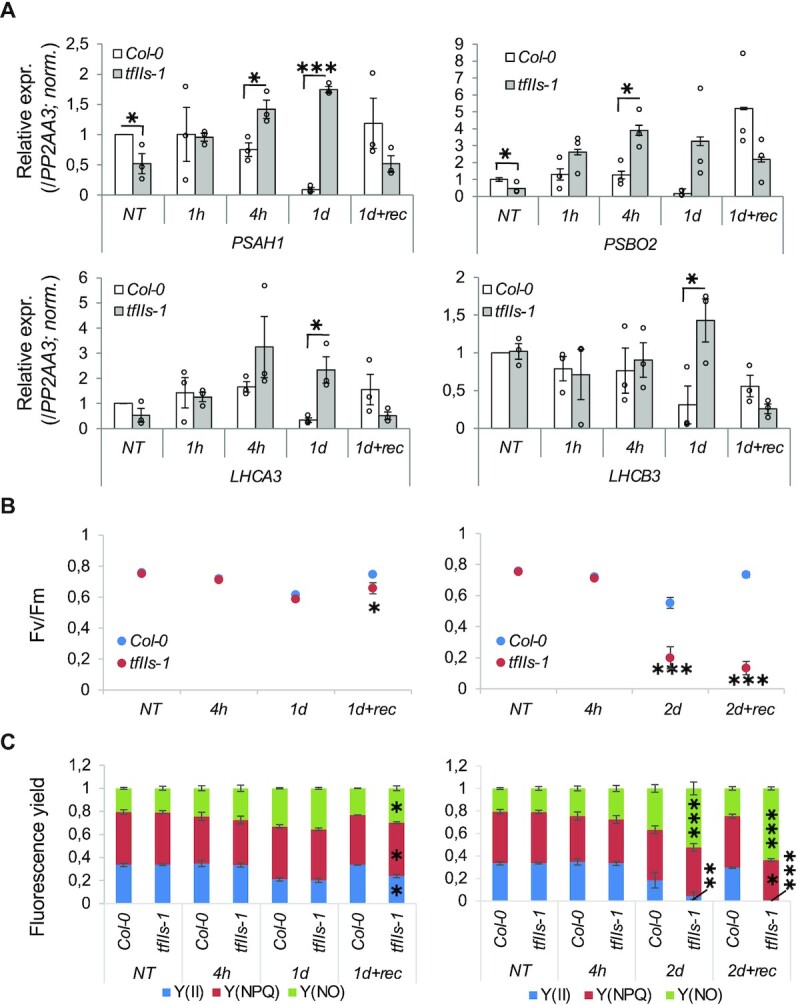
Expression of photosynthesis-related transcripts and photosynthetic activity of Col-0 and *tfIIs-1* in response to heat. Relative expression values of selected transcripts as measured by the qRT-PCR assay (**A**); the optimal Fv/Fm (**B**) and the effective *Y*(II) quantum yield of PSII, the regulated nonphotochemical quenching *Y*(NPQ) and the nonregulated *Y*(NO) heat dissipation process (**C**) were also determined; treatments as shown in Figure 4. Bars represent standard errors based on at least three biological replicates; significant alteration versus NT is not indicated; significant differences between genotypes are indicated by *P*-values based on two-tailed Student’s *t*-test (*P < 0.05, **P < 0.01, ***P < 0.001).

To specifically visualize photosystem-related transcript changes genome-wide, we collected expression values (FPKM) of transcripts encoding PSII and PSI core (Supplementary Figure S16), and light harvesting antenna complex LHCA and LHCB components (Supplementary Figure S17). Consistent with GO term analysis, a significant downregulation of all PSII, PSI, LHCA and LHCB transcripts was observed in Col-0, when plants were exposed to persistent heat, while in the *tfIIs-1* mutant these transcripts were much moderately repressed (significantly different from Col-0/1d). A mild downregulation of these mRNAs was already detected at the early stage of heat (1 h) but only in Col-0 plants. We also examined the changes of selected transcripts by qRT-PCR (Figure 11). In Col-0, the level of PSAH1, PSBO2, LHCA3 and LHCB3 transcripts was dramatically repressed, reaching a minimum at 1 day but was efficiently restored following the recovery (at 1d + rec); oppositely, in *tfIIs-1* the level of these transcripts had mostly a continuously increasing trend upon heat and dropped after the recovery period.

Next, to study the impact of heat on the function of photosynthetic apparatus, fluorescence parameters were determined in Col-0 and mutant plants (Figure 11B and C). The Fv/Fm and *Y*(II) parameters reflecting the potential and actual photosynthetic capacity of PSII often decrease under stress conditions; meanwhile, the regulated and/or nonregulated heat dissipation increases. Similarly in our experiments, the Fv/Fm and *Y*(II) values were decreased in both Col-0 and mutant plants following TMHT/1d treatment, but Fv/Fm and *Y*(II) values reverted close to the physiological levels as plants were allowed to recover for 2 days (Figure 11B and C, left panels). Col-0 plants recovered slightly better, in accordance with phenotypic observations (Figure 3). TMHT/2d treatment resulted in significantly lower Fv/Fm and *Y*(II) values in *tfIIs-1* plants (Figure 11B and C, right panels). Although the *Y*(II) decreased significantly after TMHT/2d treatment (versus NT), the regulated heat dissipation processes indicated by *Y*(NPQ) did not change significantly; the *Y*(NO) increase was accompanied by a similar Fv/Fm decrease. These results indicate that the photosynthetic apparatus suffered an irreversible injury in *tfIIs-1* plants after 2 days of heat treatment. Physiological data are in close correlation with the enhanced proteotoxic stress (Figure 10) and phenotypic observations (Figure 3). These findings suggest that the higher level of photosynthesis-related mRNA transcripts in mutant plants (Figure 11A and Supplementary Figures S16 and S17) cannot compensate the heat-induced damage of the photosynthetic apparatus to maintain photosynthetic capacity at the levels observed in Col-0 plants (Figure 11B and C).

In summary, our data show that TFIIS is needed for global transcriptome and AS pattern modulation, indirectly preventing proteotoxic stress and impairment of physiological processes, to ensure efficient heat adaptation.

## DISCUSSION

Despite the fact that TFIIS was discovered 45 years ago (83), little is known about its regulation and functions during stress responses. Here, we provide comprehensive evidence that TFIIS is an essential component of thermal adaptation in plants.

### TFIIS is dynamically regulated in response to temperature changes

Although high temperature suppresses general transcription in *Arabidopsis*, we found that it leads to rapid and dramatic upregulation of *TFIIS* mRNA, which results in enhanced TFIIS protein levels (Figure 1 and Supplementary Figure S1). It is likely that TFIIS accumulation is a consequence of transcriptional activation during heat. This conclusion is supported by the findings that (i) the levels of both unspliced and spliced forms of *TFIIS* transcripts are elevated at high temperatures, (ii) TFIIS locus contains multiple HSE *cis* elements and (iii) the TFIIS heat induction is abolished in the absence of HsfA1 transcription factors. As the TFIIS homologue of the aquatic unicellular alga *C. reinhardtii* and the TFIIS genes of dicot *B. napus* and monocot *H. vulgare* harbour HSEs and are also heat inducible (Figure 2), we propose that heat induction of TFIIS is an ancient feature, and likely occurred also in the common ancestors of green algae and plants.

Our data raise the possibility that TFIIS locus could be autoregulated: in the absence of TFIIS protein, the heat activation of TFIIS transcription is delayed (1–4 h), while in the later phase of heat exposure (1 day) the decrease of *TFIIS* mRNA does not occur but is even further elevated. The positive feedback regulation seems to be a direct effect, as TFIIS is physically bound to its own locus (Figure 1F). Transcriptional repression of TFIIS during late HS (1 day) and recovery may be the consequence of a negative feedback through TFIIS indirect action, or simply results from the ceasing of the heat activation. Supporting autoregulation hypothesis, we studied the earlier published Pol II ChIP-seq data performed from dominant negative TFIIS (*TFIISmut*)-expressing plants (6): in the presence of *TFIISmut*, the Pol II elongating complexes are enriched within the TFIIS gene body, suggesting that functional TFIIS is required for resolution of the arrested transcriptional state within the locus. In sum, the simplest explanation for these observations is that TFIIS locus is autoregulated; however, this needs to be further studied in more detail.

Thermal induction of TFIIS suggests that it is not constantly part of the elongating RNAPII, but most likely binds to RNAPII upon arrest. This idea is supported by the altered conformation of backtracked/stalled Pol II complex (4), and the two distinct conformations of TFIIS (active/open conformation when bound to Pol II and inactive/closed in solution) (84). It was suggested that TFIIS might still bind to Pol II in an inactive form to support multiple rounds of cleavage reactions per TFIIS association–dissociation cycle (84). Notably, the general transcriptome is rather suppressed in the early phase of heat exposure (Figure 4C), but TFIIS, a positive regulator of transcription, is induced (Figures 1 and 2). This suggests that the stochiometric excess of TFIIS is even more pronounced. Elevating TFIIS level therefore may (i) increase the chance of finding and binding to the arrested RNAPII or (ii) increase the TFIIS’s time spent in a Pol II-bound form, both mechanisms promoting a faster resolution of arrested RNAPII state.

### TFIIS plays a critical role in heat-induced transcriptional reprogramming

TFIIS is expressed across the whole plant body (5), and is needed for thermal adaptation during both seedling/vegetative and reproductive stages of plants’ life cycle (Figure 3 and Supplementary Figure S4). The requirement of TFIIS in the diverse HS regimes suggests that its role is not limited to certain pathways of HSR. Comparative transcriptome analysis of heat-stressed *tfIIs-1* and Col-0 plants indeed supports this hypothesis and suggests that TFIIS is required for the efficient transcriptome shift to a heat-adaptive programme: (i) transcriptional induction of HSR genes was efficient in Col-0 but was delayed in the *tfIIs-1* mutant and (ii) the transcripts involved in growth and development (including hormonal pathway, photosynthesis-linked transcripts, cell wall biosynthesis, etc.) were specifically downregulated in the Col-0 but maintained or even induced in *tfIIs-1* plants. Notably, TFIIS is not a general anti-stress factor since it was needed for high-temperature adaptation but not for salt tolerance (Supplementary Figure S6).

The lack of TFIIS may impact heat-induced transcriptome both directly and indirectly. The most important role of TFIIS is to rescue the stalled RNAPII complexes, thereby promoting efficient transcriptional elongation. Indeed, TFIIS is recruited to HS-induced genes (HSFs, HSPs) in a heat-dependent manner (Figure 7), suggesting a direct involvement in their efficient transcription.

Besides quantitative changes in transcript levels, we have shown that TFIIS is also required for proper splicing. By comparing the splicing pattern of heat-treated Col-0 and *tfIIs-1* mutant plants, hundreds of TFIIS-dependent heat-induced AS isoforms were identified. We examined these transcripts and have shown that IR events are the most prevalent forms of AS, most of the IRs being skipped in *tfIIs-1* mutant plants causing (cryptic) intron excisions. The kinetic coupling model could explain this type of change; in *tfIIs-1*, the RNAPII transcriptional processivity is low, allowing more efficient usage of weak/cryptic splice sites (15,16). However, our results suggest an indirect or alternative effect of TFIIS on splicing during heat: (i) the massive differences in splicing patterns appear prominently at 1 day heat treatment stage; (ii) the splicing pattern of U2AF65A, a highly conserved constitutive U2 splicing factor itself, is changed in the mutant; (iii) binding of TFIIS to differentially spliced loci is not heat dependent; and (iv) heat-induced intron splicing tends to be more efficient on transcripts with higher expression (Supplementary Figure S14). Interestingly, IR event changes/cryptic splicing in *tfIIs-1* plants were observed on longer genes (3.9 kb, gene length average being 2.4 kb). This might suggest that shorter transcription units are more efficiently transcribed by RNAPII in the absence of TFIIS. This is supported by the more efficient early transcription of sHSPs in comparison with longer genes/transcripts, such as HSP90 or HSP101 (Figure 6). It is also puzzling that RNAPII stall sites were enriched around the first nucleosome in the presence of *TFIISmut* (6), but specific (cryptic) intron splicing in *tfIIs-1* plants is observed downstream, at a distance of ∼2 kb from TSS, mostly during persistent heat. TFIIS’s impact on RNAPII elongation speed/processivity may indirectly alter the complex interaction between cotranscriptional RNA maturation, IR events and potential chromatin modifications. Mechanistic understanding of TFIIS on elongation process and the impact of elevated temperature on this, therefore, are far from being complete. Although the physiological relevance of TFIIS-dependent AS in heat adaptation was not studied, it can be potentially important: as most of these splicing events occur in the coding regions and could lead to the production of heat-dependent new protein isoforms required for adequate HSR. Alternatively, the novel/cryptic introns may lead to the production of nonsense transcripts (containing premature stop codon). The nonsense transcripts originated following AS events in the absence of TFIIS during heat are probably efficiently degraded by nonsense-mediated decay (NMD) and perhaps other RNA quality control mechanisms (85,86). Notably, we found no indication in the literature that NMD operates less efficiently under elevated temperatures. By examining the expression of known NMD target genes in our RNA transcriptome data (SMG7, EDS1, PR1, PR5, At5g43403, At1g22403), we could not detect significant differences between the control and the heat-treated samples. In line with these observations, we could not detect AS introns resulting in nonsense transcripts as well. The systemic detection of nonsense transcripts caused by the absence of TFIIS would be possible in an NMD-defective background (e.g. double mutant).

### TFIIS is required for proper proteostasis

We show that proteostasis is impaired in the absence of TFIIS. This conclusion is supported by the findings that in the heat-stressed *tfIIs-1* mutant (i) the expression of HSPs is severely altered, (ii) SUMO1 and ubiquitin conjugates accumulate to high levels in the early phase of heat response and (iii) increased amounts of insoluble proteins can be detected during persistent heat. This latter observation *de facto* certifies that persistent heat stress leads to elevated proteotoxicity in *tfIIs-1* mutants.

The sHSPs are the first line of defence against misfolded/unfolded protein accumulation and aggregation during stress conditions (45). We observed that particularly cytoplasmic sHSP class I (HSP17.4A, HSP17.6B, HSP17.6C, HSP18.1) transcript expression was maintained during persistent stress in *tfIIs-1*, suggesting that proteostasis stress is especially strong in the cytoplasm. Based on the chaperone titration model, the HSFs are activated and remain active until the HSPs are titrated away by binding to the denatured proteins within the cell. Interestingly, in the *tfIIs-1* mutants, the level of the ATP-dependent HSP70, HSP90 and HSP101 was relatively well suppressed, while transcriptional repression of the ATP-independent sHSPs was less pronounced or did not happen during persistent heat. Thus, the expression of the ATP-dependent and ATP-independent chaperones seems to be uncoupled in *tfIIs-1* plants. Our data suggest that perhaps sHSP proteins may be required for transcriptional repression of their own transcription and/or that their sustained induction may be upheld by the vast amount of insoluble proteins. Denatured proteins are bound by sHSP to block irreversible aggregation (47,49). At persistent heat, the amount of insoluble proteins was found to be significantly higher in *tfIIs* plants (Figure 10). The higher amount of aggregates was associated with a lower level of ubiquitination, suggesting that the ubiquitination pathway itself might be impaired in *tfIIs-1* mutants, or that ubiquitination of larger aggregates is less efficient due to the lack of accessible ubiquitination sites. In summary, the failure to refold or degrade denaturated proteins could explain the fact that the amount of insoluble protein aggregates was found to be significantly higher in *tfIIs* plants (Figure 10). We propose that the proteostasis defect in the mutant is the consequence of the inefficient transcriptional reprogramming during heat stress caused by (i) the delayed expression of HSPs (and other factors required) *en masse* and (ii) altered AS that could further lead to production of improperly folded proteins or unneeded protein isoforms.

### TFIIS is required to protect photosynthetic machinery under heat stress

We have shown that downstream physiological processes are also severely corrupted in heat-treated *tfIIs-1* mutant plants: the primary charge separation capacity and the efficiency of photosynthetic energy conversion of PSII were strongly reduced in *tfIIs-1* plants when exposed to TMHT/2d heat treatment, as shown by the changes of Fv/Fm and *Y*(II) parameters (Figure 11). The regulated heat dissipation processes manifested in *Y*(NPQ) were not activated, thus could not provide protection against heat stress. Interestingly, the photosynthetic apparatus transcripts were not downregulated efficiently in *tfIIs-1* plants, but even further induced: this may be caused by altered transcriptional regulation (e.g. repression of developmental/housekeeping pathways is impaired, observed also on wide scale by GO term enrichment); alternatively, indirect/compensatory feedback mechanisms may be at play to aid replacement of photosynthetic apparatus and to re-establish energy levels in the *tfIIs-1* plants (50).

### Efficient transcriptional activation is an essential component of HSR

Multiple TEFs, including FACT, SPT4–SPT5 (DSIF complex), SPT6, SPT6L, PAF1c, P-TEFb, TFIIF and elongator complex, act in concern to regulate elongation of RNAPII (87,88), several being actively required for heat-induced changes in a variety of organisms, including yeast (89–91), flies (92–95) or human cells (96). Our data show that in *A. thaliana* the function of TFIIS during heat stress adaptation cannot be compensated by other TEFs or TFIIS-like domain containing distant relatives. Therefore, different RNAPII auxiliary factors are needed in parallel to properly orchestrate the different aspects of elongation (e.g. chromatin remodelling, catalytic properties of Pol II or *trans* factor binding) during environmental temperature changes. How the different TEFs interact during heat stress conditions remains an exciting research opportunity.

The catalytic site of RNAPII evolved to have an incomplete nuclease core, which makes it prone to backtracking and/or stalling. The highly conserved DE motif of domain III in TFIIS completes the nuclease core of Pol II (4). This set-up therefore may deliberately allow temporary elongation halts at specific places within target loci to provide time window for cotranscriptional events and regulation of gene expression (14). What are the *cis* and *trans* factors that define target genes prone to RNAPII stalling/backtracking, and how environmental conditions affect the process is not known. In yeast, RNAPII backtracking is uniformly distributed throughout gene bodies (97). In flies and humans, backtracking sites were mapped to first nucleosomes and promoter-proximal sites, while in yeast and human cells TFIIS accumulated also downstream to polyadenylation sites suggesting functions during the transcriptional termination process. Nucleotide composition of genomic DNA is an important defining *cis* feature that regulates stalling/backtracking, having a strong preference for a T at +1 site and a weak bias for a G-stretch upstream of pause sites (14,98). In plants, RNAPII pauses at +1 nucleosome in the presence of *TFIISmut*, suggesting that passage of the first nucleosome is a particularly critical step (6). It was shown that persistent heat leads to nucleosome loss (99). This suggests that besides nucleosomes, other *cis* or *trans* factors may be involved in RNAPII stalling regulation. TFIIS ChIP experiments could help to determine the backtracking-prone sites and direct target genes during elevated heat. Besides, the TFIIS ChIP in combination with Pol II ChIP-seq data can help discriminate between TFIIS-dependent and -independent Pol II arrest-prone sites.

Conditions of high transcriptional activation may create a situation in which multiple RNAPII complexes are elongating along the gene body, especially in long genes. An arrested RNAPII complex during such conditions may inhibit multiple transcriptional complex units located upstream to the arrest site. Our results show that during heat stress in the absence of TFIIS several HSF and HSP genes are activated to a lower extent. This suggests that transcriptional arrests/backtrackings do occur under high transcriptional bursts/activation (100), and there is a clear necessity for the alleviation of these. Requirement of TFIIS under HS conditions, opposite to being nonessential during optimal conditions, suggests that (i) RNAPII arrests/backtracking events are more prevalent during HS, (ii) faster resolution of these events is needed for a prompt qualitative and quantitative transcriptional output and (iii) alternative pathways such as proteasomal removal of RNAPII complex (6) or alternative exonucleases are insufficient under such high demanding conditions. The anti-arrest activity of TFIIS not only frees the arrested RNAPII but also reduces the upstream RNAPII ‘traffic jam’ on transcriptionally highly active loci, enabling subsequent transcription cycles (Supplementary Figure S18).

## DATA AVAILABILITY

Raw RNA-seq data have been made available in the SRA repository (https://www.ncbi.nlm.nih.gov/sra/PRJNA729886).

## ACCESSION NUMBERS

TFIIS (At2g38560), PP2AA3 (At1g13320), ACT2 (At3g18780), HSFA1a (At4g17750), HSFA1b (At5g16820), HSFA1d (At1g32330), HSFA1e (At3g02990), HSFA2 (At2g26150), HSP18.2 (At5g59720), HSP22.0 (At4g10250), HSP70 (At3g12580), HSP90-1 (At5g52640), HSP101 (At1g74310), LHCA3 (At1g61520), LHCB3 (At5g54270), PSAH1 (At3g16140), PSBQ1 (At4g21280), CreTFIIS (Cre09g386350v5), CreRACK (Cre06g278222v5), BnaPP2AA5 (LOC106382560), BnaTFIISa (BnaA03g17870D), BnaTFIISb (BnaC03g21400D), BnaTFIISc (BnaC04g06710D), HvACT2 (Horvu2Hr1g0001540) and HvTFIIS (Horvu5Hr1g111700).

## Supplementary Material

gkac020_Supplemental_Files
