# Supplementary material for: Elongation factor TFIIS is essential for heat stress adaptation in plants
Source: Nucleic Acids Res. 2022 Jan 31;50(4):1927–50. doi: 10.1093/nar/gkac020 (PMC8886746; doi:10.1093/nar/gkac020)
Supplement: gkac020_Supplemental_Files [file gkac020_supplemental_files.zip › 3_SUPPEMENTARY fig_TFIIS_20211211.pdf]

S1

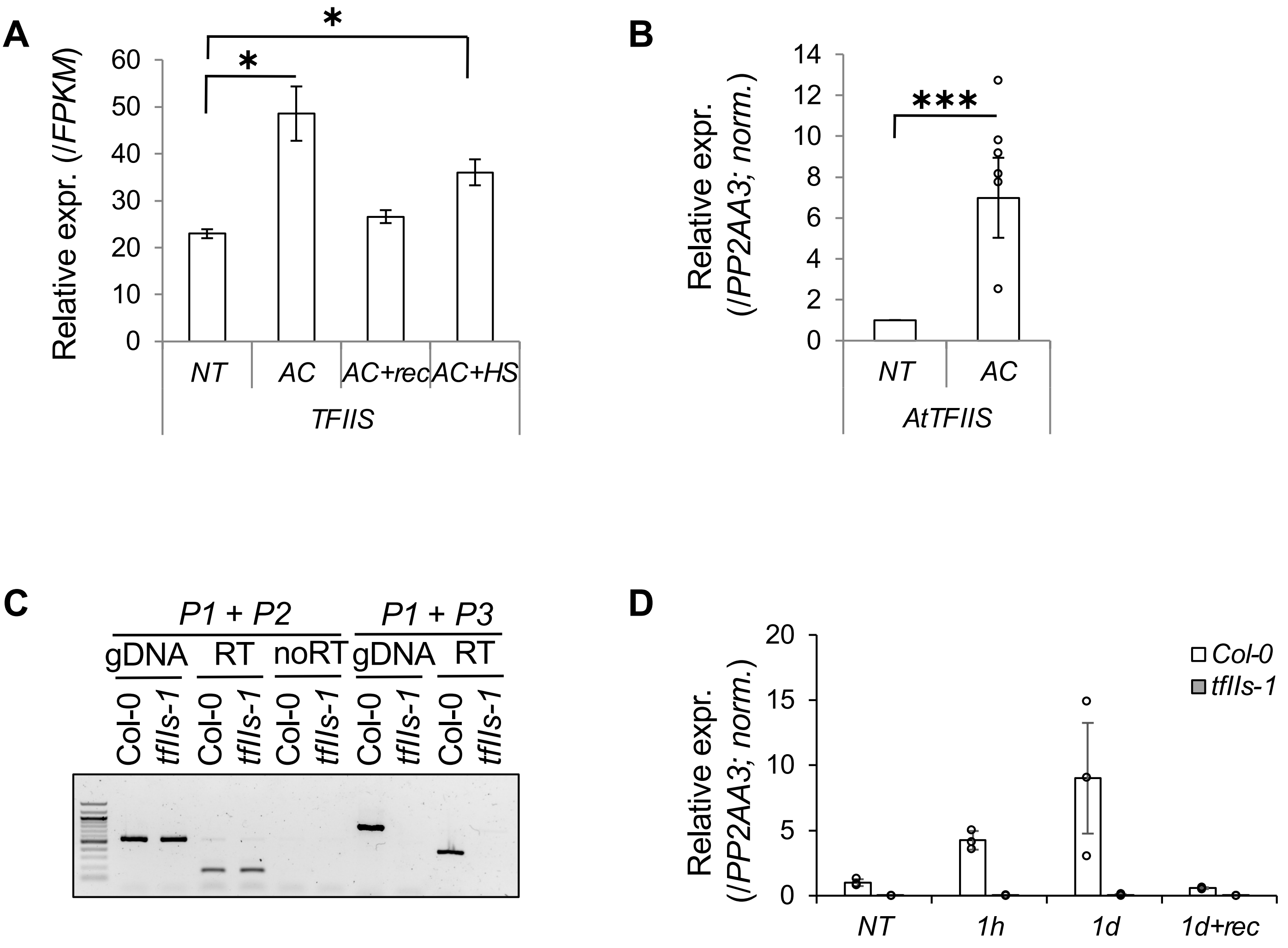

Supplementary figure 1: Expression of TFIIS is induced during heat stress. (A) Normalized FPKM value changes of *TFIIS* mRNA during non-treated (NT), acclimated (AC), recovery following acclimation (AC+rec) and heat stress following recovery and acclimation (AC+HS) based on earlier data (Szaker et al, 2019); (B) Relative expression values of *TFIIS* as measured by qRT-PCR assay in Col-0 and *tfIIs-1* plants (primers P1 and P2); (C) genotyping of *tfIIs-1* plants; (D) Relative expression of *TFIIS* transcripts as measured by qRT-PCR using primers (P1, P3) spanning the *tfIIs-1* T-DNA insertion site; Bars represent standard errors based on at least three biological replicates; p values based on two-tailed Student's t-test (*p<0.05, **p<0.01, ***p<0.001).

S2

```
                                                     CreTFIIS Domain I
AtTFIIS                ----MESDLIDLFEGAKKAADAAALDGVTSSGPEVSQCIDALKQLKKFPVTYDTLVATQVGKKLRSLAKHPVEDIKSVATDL 78
CreTFIIS               MDLISESDLNTCLKTL----ESSSQGAADGDKAEEARMLDVLKQLQRRGVTADLLKRTNAGKRLNKFCKHASDAVSKSAKAA 78
                            ****   ::      :::: ... ..  * :: :*.****::  ** * *  *:.**:*..:.**  : :.. *.

LEIWKKVVIEETAKAKKTEGTNGCKEAKVNKMDVEKPSNPAPVKVQKLQRGDSAKSIKVERKEPDNKVVTGVKIERKVPDIKVTNGTKIDYRGQAVKDEKVSKDNQ 184
VEAWKQCVKKETADGGDTAEGGASQPAK------------APMANDSAAGGREGQPLAGASAAPASG----------------TSGGGLRSAGSS--Q-------Q 154
:* **: * :***.. .*   ...: **            **:  :.   * ..: :      * .                *.*  :   *.:  :       *

                                                   CreTFIIS Domain II
SSMKAPAKAANAPPKLTAMLKCNDPVRDKIRELLVEALCRVAGEADDYERESVNASDPLRVAVSVESLMFEKLGRSTG----------AQKLKYRSIMFNLRDSNN 280
LLTRQPS--ASSSGFSVDPPRCGNETRDKVRSMLAEALAVGYVGGGDTGPSSL--QSPNQLGAAIEEALYDLMGGGGGGGGGREAVSAEYKAKARSLCFNLKDAKN 249
   : *:  *.:    .   :*.: .***:*.:*.***.     ..*   .*:  ..* ::..::*. ::: :* . *            * * **: ***:*::*

                                                CreTFIIS Domain III/Zinc-finger
PDLRRRVLTGEISPEKLITLSAEDMASDKRKQENNQIKEKALFDCERGLA-AKASTDQFKCGRCGQRKCTYYQMQTRSADEPMTTYVTCVNCDNHWKFC 378
PDLRERVLSGSVPPETLVRLSAEEMASDEQKKKNRELKEWLAKEAVRGATTNAATTDMFQCGRCKQRKCTYYQLQTRSADEPMTTFVTCTNCGQRWKFC 348
****.***:*.: **.*: ****:****::*::*.::**    :. ** :   *:** *:**** ********:**********:***.**.::****
```

Supplementary figure 2: Conservation of TFIIS protein in *Chlamydomonas reinhardtii*. Amino acid sequence alignment of *Arabidopsis thaliana* TFIIS (*At2g38560*) and *Chlamydomonas reinhardtii* TFIIS (*Cre09g386350v5*) homologous proteins: similarity between proteins is 37.84%. Locations of the conserved domains I, II and III are indicated by black bars above the sequences. Asterisks indicate conserved residues. The zinc-coordinating four cysteine residues (C; grey) and the invariant acidic hairpin residues (DE; bold, grey and underlined) within domain III are indicated. Amino acid positions within the individual sequences are given to the right.

Supplementary figure 3: Conservation of TFIIS protein in dicot *Brassica napus* and monocot crop *Hordeum vulgare*. (A) Phylogenetic relationship of the predicted TFIIS protein homologs (left) in *Brassica napus* (BnaTFIISa-c), *Brassica rapa* (Bra), *Brassica oleracea* (Bo) and *Hordeum vulgare* (HvTFIIS) proteins and similarity percent between homologous proteins (right). (B) Amino acid sequence alignment of *Arabidopsis thaliana TFIIS* (*At2g38560*) and predicted homologous proteins as shown in (A). Locations of the conserved domains I, II and III are indicated by black bars above the sequences; residues delineating the LW motif predicted to mediate nuclear localization of TFIIS are highlighted in red; the four cysteine residues (C; grey) in domain III that coordinate a zinc ion, and invariant acidic hairpin residues (DE; bold, grey and underlined); amino acid positions within the individual sequences are given on the right.

▼

S3

A

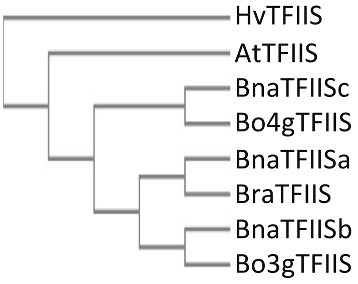

	HvTFIIS	AtTFIIS	BnaTFIISc	Bo4gTFIIS	BnaTFIISa	BraTFIIS	BnaTFIISb	Bo3gTFIIS
HvTFIIS	100.00	57.53	58.98	59.29	59.77	58.94	59.48	54.66
AtTFIIS	57.53	100.00	80.06	80.06	84.15	82.97	82.71	81.87
BnaTFIISc	58.98	80.06	100.00	98.53	86.25	84.57	85.00	83.98
Bo4gTFIIS	59.29	80.06	98.53	100.00	86.46	84.80	85.23	84.21
BnaTFIISa	59.77	84.15	86.25	86.46	100.00	99.43	97.72	98.01
BraTFIIS	58.94	82.97	84.57	84.80	99.43	100.00	97.72	97.83
BnaTFIISb	59.48	82.71	85.00	85.23	97.72	97.72	100.00	99.43
Bo3gTFIIS	54.66	81.87	83.98	84.21	98.01	97.83	99.43	100.00

B

```
AtTFIIS        -----------------------------------------------------MESDLIDLFEGAKKAADAAALDGVTSSGP 29
BnaTFIISa      -----------------------------------------------------MESELIALFETAKKAADAAAVDGVTSSSP 29
BnaTFIISb      -----------------------------------------------------MESELIALFEAAKKAADAVAVDGVTSSSP 29
BnaTFIISc      -----------------------------------------------------MESELIELFEAAKKAADAAAIDGVTCTGQ 29
BraTFIIS       -----------------------------------------------------MESELIALFETAKKAADAAAVDGLTSSSP 29
Bo3g032540     TPLSLSSPRDQNEVPPNKSRNPIFVLIPFFFNRHSPQRKLGALLSGAKRFEVTMESELIALFEAAKKAADAVAVDGVTSSSP 82
Bo4g027160     -----------------------------------------------------MESELIELFEAAKKAADAAAIDGVTSTGQ 29
HvTFIIS        ----VASEPDTREGRP--RRRGLLLRLG--FSSPPPP------PPPPPASRMAMERELMETFEAAKKAADAAAEE-ADGSPP 67
                                                                     ** :*:  ** *******.* :    :
```

Domain I

```
EVSQCIDALKQLKKFPVTYDTLVATQVGKKLRSLAKHPVEDIKSVATDLLEIWKKVVIEETAK-AKKTEGTNGCKEAKVNKMDVEKPSNPAPVKVQKLQRGDSAKS 134
EVSQSLDALEKLKAFPVTYNTLVATQVGKKLRSLAKHPVEEIKSVATDLLETWKRVVIEETTSKAKKTESTNGCKE---------------------LQRGDSAKS 114
EVSQSLDALEKLKTFPVTYNTLVATQVGKKLRSLAKHPVEEIKSVATDLLETWKRVVIEETTSKAKKTESTNGCKE---------------------LQRGDSAKS 114
EVSRSLDALKQLKKFPITYDMLVATQVGKKLRSLSKHPIDEIKTVATDLLEIWKKLVIEETSKSKL---------ERKDA----DKTPNPTPVKVQKLQRGDSAKS 122
EVSQSLDALEKLKTFPVTYNTLVATQVGKKLRSLAKHPVEEIKSVATDLLETWKRVVIEETTSKAKKTESTNGCKEVKLE----RKDVNPAPVKVQKLQRGDSAKS 131
EVSQSLDALEKLKTFPVTYNTLVATQVGKKLRSLAKHPVEEIKSVATDLLETWKRVVIEETTSKAKKTESTNGCKEVKLE----RKDVNPVPVKVQKLQRGDSAKS 184
EVSRSLDALKQLKKFPITYDMLVATQVGKKLRSLSKHPIDEIKTVATDLLEIWKKLVIEETSKSKL---------ERKDV----DKTPNPTPVKVQKLQRGDSAKS 122
EAQRCLDALRRLREFRVNTDVLVATQVGKRLRYLTKHPNSDIQAMAADLFGYWKKVVIEETGKKNGTPA--NGKSDNS-----AAKADKPQPMKVEKNSVSA---K 163
*..:.:***.:*: * :. : ********:** *:*** .:*:::*:**:  **::***** .               :                  . .    .

IKVER------KE-PDNKVVTGVKIERKVPDIKVTNGTKIDYRGQ-AVKDEKVSKDNQSSMKAPAK--AANAPPKLTAMLKCNDPVRDKIRELLVEALCRVAGEAD 230
IKVER------K-EPDSKV----KVERKEPDTKV----KMDHRGQTTVKDEKVSKETLSSVKA--SDKAPNGAPKLTSMVKCNDPVRDKIRELLVDAMSKVHGESD 203
IKVER------K-EPDSKV----KVERKEPDNKV----KMDHRGQTTVKDEKVSSETLTSVKP--SDKAPNGAPKLTSMVKCNDPVRDKIRELLVDAMSKVHGESD 203
IKVEE------RKERDNKV---------NAGA--------------------SKENQSSMKAPAKAPSTNGAPKLTSMVKCNDPVRDKIRELLVDAMSKVHDESD 192
IKVER------K-EPDSKV----KVERKEPDTKV----KMDHRGQTTVKDEKVSKETLSSVKA--SDKAPNGAPKLTSMVKCNDPVRDKIRELLVDAMSKVHGESD 220
IKVER------K-EPDSKV----KVERKEPDNKV----KMDHRGQTTVKDEKVSRETLTSVKP--SDKAPNGAPKLTSMVKCNDPVRDKIRELLVDAMSKVHGESD 273
IKVEE------RKERDNGV----KVERKDAGA--------------------SKENQSSMKAPAKAPSNNGAPKLTSMVKCNDPVRDKIRELLVDAMSKVHDESD 197
VKIEKNSTSATVKTEKSSMSTSVKIEKKDGSIKVEKTENND--SKVQVKVEKVSKEVSRTPDTKKQSSVPNGPPKLTSLVRCNDAARDKYRELLVEAFVKVSKETS 267
:*:*.           .. :           .                     * :   : .   .    *. ****::::*** .*** *****:*: :*  *:.
```

Domain II

```
DYERE-------SVNASDPLRVAVSVESLMFEKLGRSTGAQKLKYRSIMFNLRDSNNPDLRRRVLTGEISPEKLITLSAEDMASDKRKQENNQIKEKALFDCERGL 329
EYDRA-------RVVGCDPIRVAVSVESHMFEKLGRSTGAQKVKYRSIMFNLRDSNNPDLRRRVLTGEVSPEKLITLSGEEMASDKRKQETNQIKEKFLFDCERGQ 302
EYDRA-------RVVGCDPIRVAVSVESHIFEKLGRSTGAQKVKYRSIMFNLRDSNNPDLRRRVLTGEVSPEKLITLSGEEMASDKRKQETNQIKEKFLFDCERGQ 302
DYDRA-------RVVGCDPIRVAVSVESHMFEKLGRSTGAQKVKYRSIMFNLRDSNNPDLRRRVLTGEVSPEKLITLSGEEMASDKRKQETNQIKEKFLFDCERGQ 291
EYDRA-------RVVGCDPIRVAVSVESHMFEKLGRSTGAQKVKYRSIMFNLRDSNNPDLRRRVLTGEVSPEKLITLSGEEMASDKRKQETNQIKEKFLFDCERGQ 319
EYDRA-------RVVGCDPIRVAVSVESHMFEKLGRSTGAQKVKYRSIMFNLRDSNNPDLRRRVLTGEVSPEKLITLSGEEMASDKRKQETNQIKEKFLFDCERGQ 372
DYDRA-------RVVGCDPIRVAVSVESHMFEKLGRSTGAQKVKYRSIMFNLRDSNNPDLRRRVLTGEVSPEKLITLSGEEMASDKRKQETNQIKEKFLFDCERGQ 296
EDDREEIRDLLNQVNACDPYRVAVTVESALFERIGRSTGTHKVKYRSILFNLKADNNPDFRRRVLLGEVRPGSLVDMPADEMASDARKLENQQIKEKALFDCERAS 373
: :*          * ..** ****:*** :**::*****::*:*****:***: .****:***** **: * .*: : .::**** ** *.:***** ******.
```

Domain III/Zinc-finger

```
AAKASTDQFKCGRCGQRKCTYYQMQTRSADEPMTTYVTCVNCDNHWKFC 378
APKASTDQFKCGRCGQRKCTYYQMQTRSADEPMTTYVTCVNCDNHWKFC 351
APKASTDQFKCGRCGQRKCTYYQMQTRSADEPMTTYVTCVNCDNHWKFC 351
APKASTDQFKCGRCGQRKCTYYQMQTRSADEPMTTYVTCVNCDNHWKFC 340
APKASTDQFKCGRCGQRKCTYYQMQTRSADEPMTTYVTCVNCDNHWKFC 368
APKASTDQFKCGRCGQRKCTYYQMQTRSADEPMTTYVTCVNCDNHWKFC 421
APKASTDQFKCGRCGQRKCTYYQMQTRSADEPMTTYVTCVNCDNHWKFC 345
APKASTDQFKCGRCGQRKTTYYQLQTRSADEPMTTFVTCVNCNNHWKFC 422
* *************** ****:***********:******:******
```

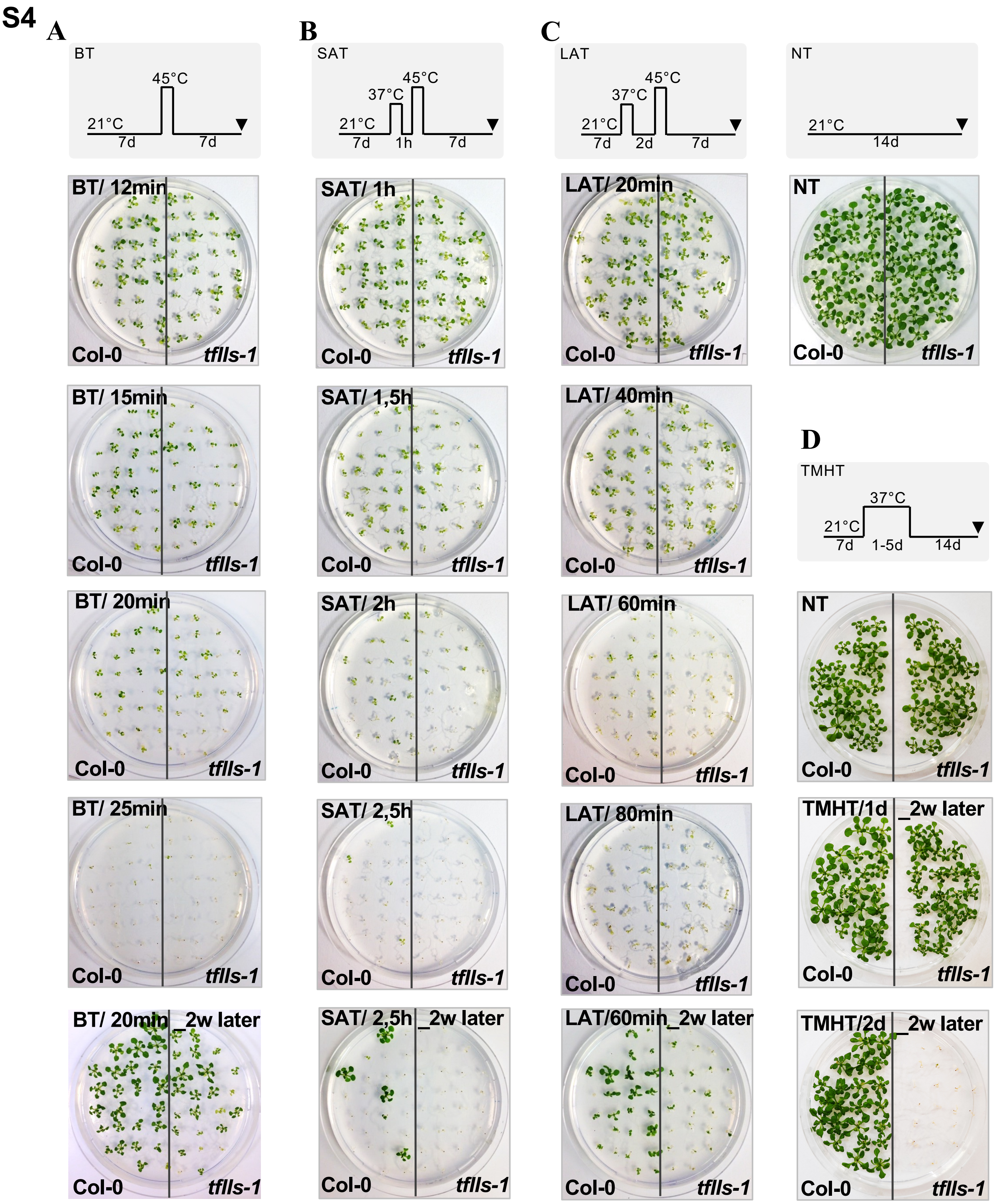

Supplementary figure 4: TFIIS is needed for heat stress adaptation during different heat stress thermotolerance regimes. Col-0 and *tfIIs-1* plant were subjected to (A) basal thermotolerance (BT), (B) short acquired thermotolerance (SAT), (C) long acquired thermotolerance (LAT) or (D) thermotolerance to moderately high temperatures (TMHT) heat stress regimes for the indicated times; pictures were taken one or two weeks after treatments. Schematic representation of heat stress regimes are summarized at the top of each.

S5

A

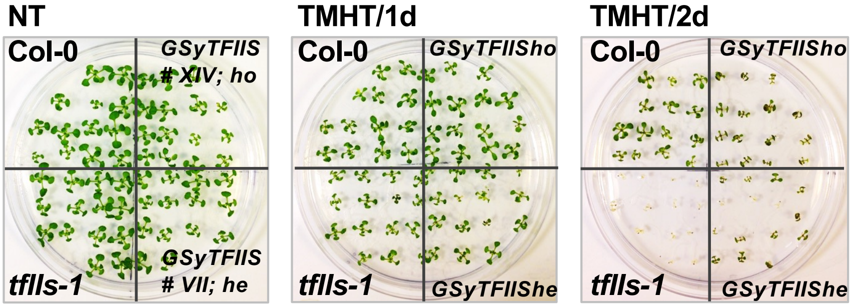

B

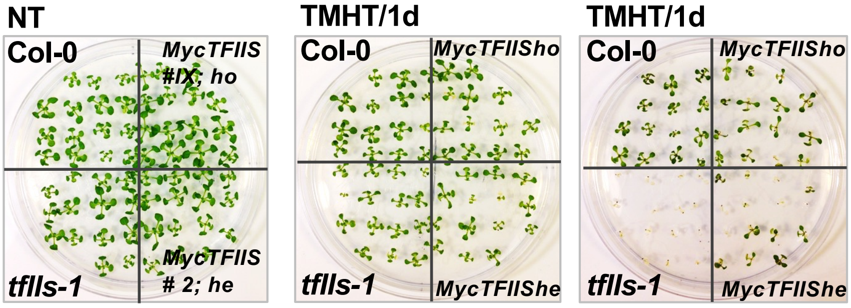

Supplementary figure 5: Tagged TFIIS constructs complement the heat-sensitive phenotype of *tfIIs-1* mutant plants. Col-0, *tfIIs-1* mutant and (A) *pTFIIS::GSy-TFIIS;tfIIs-1* or (B) *p35S::2xMyc-TFIIS;tfIIs-1* plant were subjected to Thermotolerance to Moderately High Temperature (TMHT) regime for one (1d) or two days (2d) as indicated; pictures were taken one week after treatments.

S6

A

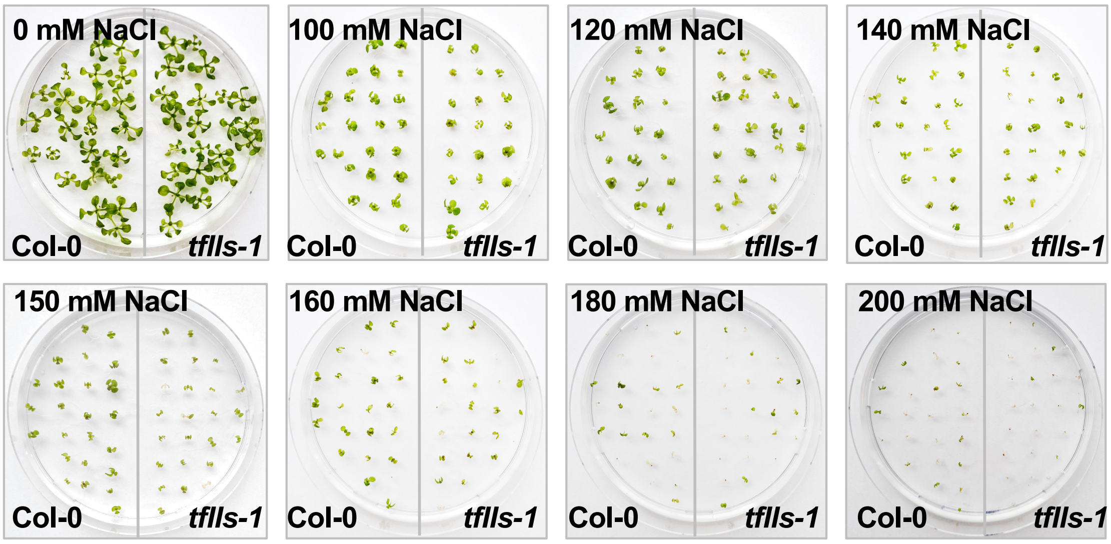

B

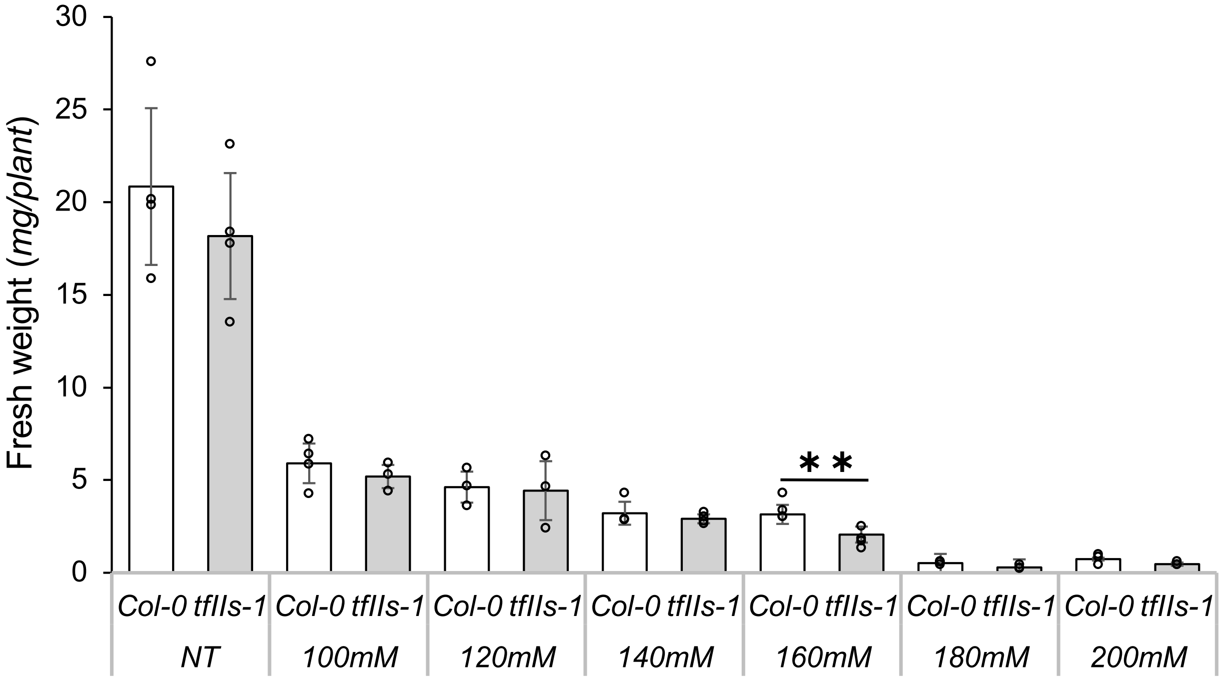

Supplementary figure 6: Salt stress tolerance of wild type and tfIIs-1 mutant plants. (A) Col-0 and *tfIIs-1* plants were grown on different salt concentrations as indicated, pictures were taken at 14 days; (B) Fresh weight of Col-0 and tfIIs-1 mutant green seedlings was measured at the end of salt treatment (14 days); bars represent standard errors based on at least three biological replicates; p values based on two-tailed Student's t-test (*p<0.05, **p<0.01, ***p<0.001).

S7

NT vs 1h
(2058 genes; best 5 terms/source)

source	term_name	term_id	adjusted_p_value
GO:MF	electron transporter, transferring electrons within the cyclic electron transport pathway of photosynthesis activity	GO:0045156	0.000335363
GO:MF	squalene monooxygenase activity	GO:0004506	0.022898108

source	term_name	term_id	adjusted_p_value
GO:MF	unfolded protein binding	GO:0051082	8.08E-30
GO:MF	heat shock protein binding	GO:0031072	1.11E-17
GO:MF	misfolded protein binding	GO:0051787	5.68E-14
GO:MF	protein binding	GO:0005515	3.13E-08
GO:MF	protein folding chaperone	GO:0044183	3.20E-08
GO:BP	response to abiotic stimulus	GO:0009628	4.44E-61
GO:BP	response to heat	GO:0009408	2.86E-50
GO:BP	cellular response to hypoxia	GO:0071456	5.96E-47
GO:BP	cellular response to decreased oxygen levels	GO:0036294	1.33E-46
GO:BP	cellular response to oxygen levels	GO:0071453	1.33E-46
GO:CC	intracellular anatomical structure	GO:0005622	6.31E-21
GO:CC	cytoplasm	GO:0005737	4.13E-19
GO:CC	intracellular membrane-bounded organelle	GO:0043231	7.64E-16
GO:CC	membrane-bounded organelle	GO:0043227	4.23E-15
GO:CC	intracellular organelle	GO:0043229	3.36E-14

source	term_name	term_id	adjusted_p_value
GO:CC	proton-transporting ATP synthase complex, coupling factor F(o)	GO:0045263	0.046885085

source	term_name	term_id	adjusted_p_value
GO:MF	structural constituent of cell wall	GO:0005199	5.5726E-06
GO:MF	oxidoreductase activity	GO:0016491	0.000199997
GO:BP	response to oxidative stress	GO:0006979	4.11949E-05
GO:BP	oxidation-reduction process	GO:0055114	0.000482552
GO:BP	response to toxic substance	GO:0009636	0.000510693
GO:BP	detoxification	GO:0098754	0.002494438
GO:BP	plant-type cell wall organization	GO:0009664	0.004959366
GO:CC	secretory vesicle	GO:0099503	0.009632846
GO:CC	anchored component of membrane	GO:0031225	0.023487975

source	term_name	term_id	adjusted_p_value
GO:MF	peroxidase activity	GO:0004601	1.26006E-05
GO:MF	oxidoreductase activity, acting on peroxide as acceptor	GO:0016684	1.92353E-05
GO:MF	oxidoreductase activity	GO:0016491	3.51437E-05
GO:MF	antioxidant activity	GO:0016209	8.27364E-05
GO:MF	chitinase activity	GO:0004568	0.000571898
GO:BP	hydrogen peroxide catabolic process	GO:0042744	1.13519E-05
GO:BP	cell wall organization or biogenesis	GO:0071554	7.65836E-05
GO:BP	hydrogen peroxide metabolic process	GO:0042743	8.78843E-05
GO:BP	cellular detoxification	GO:1990748	0.000854299
GO:BP	cellular response to toxic substance	GO:0097237	0.000899305
GO:CC	anchored component of membrane	GO:0031225	3.82E-08
GO:CC	extracellular region	GO:0005576	5.24E-08
GO:CC	plant-type cell wall	GO:0009505	6.07E-08
GO:CC	cell wall	GO:0005618	3.69E-07
GO:CC	external encapsulating structure	GO:0030312	3.89E-07

source	term_name	term_id	adjusted_p_value
GO:MF	transporter activity	GO:0005215	0.001068538
GO:MF	transmembrane transporter activity	GO:0022857	0.001185272
GO:MF	heme binding	GO:0020037	0.004839037
GO:MF	inorganic molecular entity transmembrane transporter activity	GO:0015318	0.005638626
GO:MF	peroxidase activity	GO:0004601	0.005899578
GO:BP	response to stimulus	GO:0050896	4.83435E-05
GO:BP	hydrogen peroxide catabolic process	GO:0042744	0.000505598
GO:BP	response to toxic substance	GO:0009636	0.001062018
GO:BP	secondary metabolic process	GO:0019748	0.001519224
GO:BP	response to stress	GO:0006950	0.001978259
GO:CC	cell periphery	GO:0071944	2.66E-10
GO:CC	plant-type cell wall	GO:0009505	1.09725E-05
GO:CC	plasma membrane	GO:0005886	1.26047E-05
GO:CC	external encapsulating structure	GO:0030312	5.84879E-05
GO:CC	cell wall	GO:0005618	0.000216341

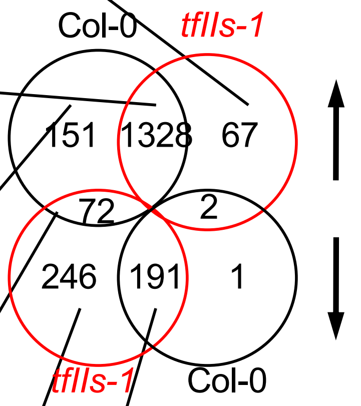

Supplementary figure 7: Gene ontology (GO) term analysis of differentially expressing genes (DEGs) between non-treated and one hour heat stress (37°C/1h). Best 5 terms/source are shown, molecular function (MF), biological process (BP), cell compartment (CC); terms ID numbers and adjusted significance p values are listed for each; numbers of up- or down-regulated DEGs in Col-0 and *tfIIs-1* are shown (right); Col-0 (black), *tfIIs-1* mutant (red).

S8 *NT vs 1d (1069 genes; best 5 terms/source)*

source	term_name	term_id	adjusted_p_value
GO:MF	RNA binding	GO:0003723	9.55E-122
GO:MF	mRNA binding	GO:0003729	8.85E-78
GO:MF	structural constituent of ribosome	GO:0003735	1.18E-73
GO:MF	structural molecule activity	GO:0005198	2.87E-63
GO:MF	unfolded protein binding	GO:0051082	1.28E-33
GO:BP	ribonucleoprotein complex biogenesis	GO:0022613	2.28E-98
GO:BP	RNA processing	GO:0006396	5.23E-88
GO:BP	translation	GO:0006412	2.40E-75
GO:BP	peptide biosynthetic process	GO:0043043	1.46E-74
GO:BP	peptide metabolic process	GO:0006518	4.76E-72
GO:CC	intracellular anatomical structure	GO:0005622	8.94E-152
GO:CC	protein-containing complex	GO:0032991	1.77E-144
GO:CC	ribonucleoprotein complex	GO:1990904	2.52E-144
GO:CC	intracellular organelle	GO:0043229	2.67E-141
GO:CC	organelle	GO:0043226	1.84E-135

source	term_name	term_id	adjusted_p_value
GO:MF	peptide-methionine (R)-S-oxide reductase activity	GO:0033743	0.013423942
GO:MF	ureidoglycolate hydrolase activity	GO:0004848	0.020019875
GO:BP	response to abiotic stimulus	GO:0009628	3.80E-07
GO:BP	protein repair	GO:0030091	0.003970032
GO:BP	response to high light intensity	GO:0009644	0.021610365
GO:BP	response to light intensity	GO:0009642	0.04515677
GO:BP	macromolecule localization	GO:0033036	0.048780161
GO:CC	plastid envelope	GO:0009526	1.88E-07
GO:CC	chloroplast envelope	GO:0009941	6.76E-07
GO:CC	endomembrane system	GO:0012505	3.66139E-06
GO:CC	cytoplasm	GO:0005737	5.1023E-06
GO:CC	organelle envelope	GO:0031967	8.86308E-06

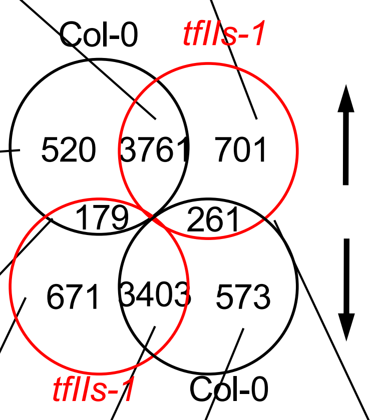

source	term_name	term_id	adjusted_p_value
GO:MF	nucleic acid binding	GO:0003676	9.1365E-05
GO:MF	RNA binding	GO:0003723	0.007033452
GO:MF	protein methyltransferase activity	GO:0008276	0.009241183
GO:MF	catalytic activity, acting on RNA	GO:0140098	0.026389295
GO:MF	N-methyltransferase activity	GO:0008170	0.045285154
GO:BP	nucleobase-containing compound metabolic proc	GO:0006139	2.53E-09
GO:BP	heterocycle metabolic process	GO:0046483	3.06E-09
GO:BP	cellular nitrogen compound metabolic process	GO:0034641	1.61E-08
GO:BP	ncRNA processing	GO:0034470	3.29E-08
GO:BP	organic cyclic compound metabolic process	GO:1901360	4.06E-08
GO:CC	nuclear protein-containing complex	GO:0140513	2.59E-08
GO:CC	intracellular anatomical structure	GO:0005622	4.93E-08
GO:CC	mitochondrion	GO:0005739	1.54E-07
GO:CC	intracellular organelle	GO:0043229	4.54E-07
GO:CC	intracellular membrane-bounded organelle	GO:0043231	7.57E-07

source	term_name	term_id	adjusted_p_value
GO:MF	catalytic activity, acting on RNA	GO:0140098	0.000952388
GO:BP	macromolecule metabolic process	GO:0043170	0.000729325
GO:BP	gene silencing	GO:0016458	0.001902679
GO:BP	nucleic acid metabolic process	GO:0090304	0.008925158
GO:BP	RNA metabolic process	GO:0016070	0.01553134
GO:BP	nitrogen compound metabolic process	GO:0006807	0.0165614
GO:CC	nucleus	GO:0005634	0.007554624
GO:CC	intracellular anatomical structure	GO:0005622	0.01227925
GO:CC	transferase complex	GO:1990234	0.013497201
GO:CC	nuclear protein-containing complex	GO:0140513	0.034614775
GO:CC	transferase cplx, transferring phosphorus-groups	GO:0061695	0.044364034

source	term_name	term_id	adjusted_p_value
GO:MF	actin filament binding	GO:0051015	0.002396668
GO:MF	actin binding	GO:0003779	0.002518329
GO:BP	tissue development	GO:0009888	5.03364E-05
GO:BP	plant epidermis development	GO:0090558	0.0001331
GO:BP	supramolecular fiber organization	GO:0097435	0.000281416
GO:BP	cell development	GO:0048468	0.001339756
GO:BP	cytoskeleton organization	GO:0007010	0.001388531
GO:CC	cytoskeleton	GO:0005856	0.000502695
GO:CC	Golgi apparatus subcompartment	GO:0098791	0.000586663
GO:CC	actin cytoskeleton	GO:0015629	0.001004508
GO:CC	Golgi membrane	GO:0000139	0.002115023
GO:CC	vesicle tethering complex	GO:0099023	0.009367167

source	term_name	term_id	adjusted_p_value
GO:MF	protein domain specific binding	GO:0019904	2.77E-07
GO:MF	mRNA binding	GO:0003729	2.82699E-05
GO:MF	chlorophyll binding	GO:0016168	0.000431845
GO:MF	oxidoreductase activity	GO:0016491	0.001099701
GO:BP	photosynthesis	GO:0015979	1.72E-19
GO:BP	photosynthesis, light reaction	GO:0019684	3.15E-13
GO:BP	photosynthetic electron transport chain	GO:0009767	2.57E-10
GO:BP	generation of precursor metabolites and energy	GO:0006091	4.20E-08
GO:BP	photosynthetic electron transport in PS I	GO:0009773	1.04501E-05
GO:CC	chloroplast thylakoid	GO:0009534	3.74E-43
GO:CC	plastid thylakoid	GO:0031976	4.20E-43
GO:CC	chloroplast thylakoid membrane	GO:0009535	6.61E-42
GO:CC	plastid thylakoid membrane	GO:0055035	7.52E-42
GO:CC	thylakoid	GO:0009579	1.22E-41

source	term_name	term_id	adjusted_p_value
GO:MF	catalytic activity	GO:0003824	9.72E-12
GO:MF	symporter activity	GO:0015293	9.35E-09
GO:MF	DNA-binding transcription factor activity	GO:0003700	2.53072E-06
GO:MF	protein kinase activity	GO:0004672	4.17184E-06
GO:BP	response to stimulus	GO:0050896	1.40E-27
GO:BP	response to abiotic stimulus	GO:0009628	1.57E-22
GO:BP	response to chemical	GO:0042221	1.23E-21
GO:BP	small molecule metabolic process	GO:0044281	2.72E-20
GO:BP	oxoacid metabolic process	GO:0043436	4.24E-18
GO:CC	cell periphery	GO:0071944	1.64E-29
GO:CC	plasma membrane	GO:0005886	7.19E-24
GO:CC	chloroplast	GO:0009507	1.53E-12
GO:CC	plastid	GO:0009536	2.30E-12
GO:CC	plant-type cell wall	GO:0009505	7.82E-11

source	term_name	term_id	adjusted_p_value
GO:MF	protein domain specific binding	GO:0019904	2.71648E-05
GO:MF	tetrapyrrole binding	GO:0046906	0.015478107
GO:MF	oxidoreductase activity, paired donors	GO:0016717	0.044961659
GO:BP	photosynthesis	GO:0015979	1.94E-07
GO:BP	photosynthesis, light reaction	GO:0019684	6.44315E-05
GO:CC	chloroplast	GO:0009507	3.50E-17
GO:CC	thylakoid	GO:0009579	6.15E-16
GO:CC	plastid	GO:0009536	9.02E-15
GO:CC	plastid thylakoid	GO:0031976	5.69E-13
GO:CC	chloroplast thylakoid	GO:0009534	2.46E-12

Supplementary figure 8: Gene ontology (GO) term analysis of differentially expressing genes (DEGs) between non-treated (NT) and one day heat stress (37°C/1d). Best 5 terms/source are shown, molecular function (MF), biological process (BP), cell compartment (CC); terms ID numbers and adjusted significance p values are listed for each; numbers of up- or down-regulated DEGs in Col-0 and *tfIIs-1* are shown (right); Col-0 (black), *tfIIs-1* mutant (red).

S9

NT vs 1d+Rec
(1628 genes; best 5 terms/source)

source	term_name	term_id	adjusted_p_value
GO:BP	response to chemical	GO:0042221	0.000124252
GO:BP	response to stimulus	GO:0050896	0.00031998
GO:BP	cellular response to chemical stimulus	GO:0070887	0.003856056
GO:BP	hormone-mediated signaling pathway	GO:0009755	0.042895446

source	term_name	term_id	adjusted_p_value
GO:MF	oxidoreductase activity	GO:0016491	3.37E-16
GO:MF	heme binding	GO:0020037	2.38E-08
GO:MF	catalytic activity	GO:0003824	3.15E-08
GO:MF	tetrapyrrole binding	GO:0046906	2.38E-07
GO:MF	glutathione transferase activity	GO:0004364	3.49E-07
GO:BP	response to stress	GO:0006950	8.99E-36
GO:BP	response to stimulus	GO:0050896	2.65E-29
GO:BP	response to external biotic stimulus	GO:0043207	9.28E-25
GO:BP	response to other organism	GO:0051707	9.28E-25
GO:BP	response to external stimulus	GO:0009605	3.15E-24
GO:CC	secretory vesicle	GO:0099503	1.57E-08
GO:CC	vacuole	GO:0005773	6.80E-07
GO:CC	extracellular region	GO:0005576	1.06E-05
GO:CC	cytosol	GO:0005829	1.42E-05
GO:CC	cell wall	GO:0005618	4.65E-05

source	term_name	term_id	adjusted_p_value
GO:MF	tetrapyrrole binding	GO:0046906	0.003110193
GO:MF	heme binding	GO:0020037	0.012656611
GO:MF	glutathione transferase activity	GO:0004364	0.013438774
GO:BP	toxin metabolic process	GO:0009404	4.18761E-05
GO:BP	detoxification	GO:0098754	0.001611436
GO:BP	sulfur compound metabolic process	GO:0006790	0.00192435
GO:BP	response to toxic substance	GO:0009636	0.004669407
GO:BP	biological process involved in interspecies interaction between organisms	GO:0044419	0.005561716

source	term_name	term_id	adjusted_p_value
GO:MF	oxidoreductase activity	GO:0016491	0.006274721
GO:MF	antioxidant activity	GO:0016209	0.007557412
GO:MF	heme binding	GO:0020037	0.042713818
GO:BP	response to toxic substance	GO:0009636	0.000197032
GO:BP	detoxification	GO:0098754	0.001093741
GO:BP	thalianol metabolic process	GO:0080003	0.011786433
GO:BP	cellular response to phosphate starvation	GO:0016036	0.022632829
GO:BP	tricyclic triterpenoid metabolic process	GO:0010683	0.023538056

source	term_name	term_id	adjusted_p_value
GO:MF	structural constituent of cell wall	GO:0005199	4.31E-08
GO:MF	heme binding	GO:0020037	1.9112E-06
GO:MF	tetrapyrrole binding	GO:0046906	9.0731E-06
GO:MF	peroxidase activity	GO:0004601	9.91307E-06
GO:MF	oxidoreductase activity, acting on peroxide as acceptor	GO:0016684	1.61441E-05
GO:BP	cell wall organization or biogenesis	GO:0071554	1.20E-09
GO:BP	cell wall organization	GO:0071555	6.60E-08
GO:BP	external encapsulating structure organization	GO:0045229	7.51E-08
GO:BP	cell-cell junction assembly	GO:0007043	1.68721E-06
GO:BP	hydrogen peroxide catabolic process	GO:0042744	3.62961E-06
GO:CC	extracellular region	GO:0005576	9.45E-09
GO:CC	Casparian strip	GO:0048226	1.12408E-06
GO:CC	cell wall	GO:0005618	1.86805E-06
GO:CC	external encapsulating structure	GO:0030312	1.98078E-06
GO:CC	plant-type cell wall	GO:0009505	7.33116E-06

source	term_name	term_id	adjusted_p_value
GO:MF	chlorophyll binding	GO:0016168	6.97E-11
GO:MF	tetrapyrrole binding	GO:0046906	4.74E-10
GO:MF	oxidoreductase activity	GO:0016491	0.002160019
GO:MF	heme binding	GO:0020037	0.014980568
GO:MF	oxidoreductase activity, acting on paired donors	GO:0016705	0.015433411
GO:BP	response to stimulus	GO:0050896	3.71E-08
GO:BP	response to chemical	GO:0042221	1.26E-07
GO:BP	protein-chromophore linkage	GO:0018298	2.02E-07
GO:BP	response to light stimulus	GO:0009416	2.75E-07
GO:BP	photosynthesis, light harvesting in photosystem I	GO:0009768	3.01E-07
GO:CC	photosystem II	GO:0009523	6.57E-09
GO:CC	plastoglobule	GO:0010287	1.50E-08
GO:CC	photosystem	GO:0009521	1.31E-07
GO:CC	photosystem I	GO:0009522	2.43277E-05
GO:CC	chloroplast thylakoid membrane	GO:0009535	0.002055051

source	term_name	term_id	adjusted_p_value
GO:BP	response to auxin	GO:0009733	1.06E-07
GO:BP	cellular response to chemical stimulus	GO:0070887	1.48442E-06
GO:BP	response to hormone	GO:0009725	2.46342E-06
GO:BP	response to endogenous stimulus	GO:0009719	3.17691E-06
GO:BP	cellular response to hormone stimulus	GO:0032870	1.11937E-05

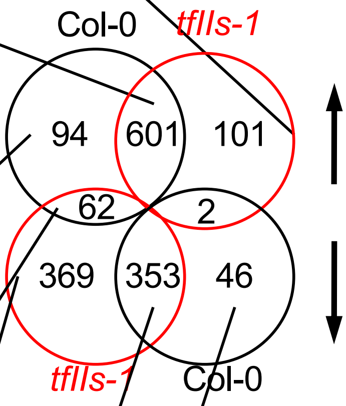

Supplementary figure 9: Gene ontology (GO) term analysis of differentially expressing genes (DEGs) between non-treated (NT) and one day heat stress followed by two days recovery period at NT (1d+rec). Best 5 terms/source are shown, molecular function (MF), biological process (BP), cell compartment (CC); terms ID numbers and adjusted significance p values are listed for each; numbers of up- or down-regulated DEGs in Col-0 and *tfIIs-1* are shown (right); Col-0 (black), *tfIIs-1* mutant (red).

S10

A

NT; Col-0 vs tfIIs-1; higher expression in Col-0 (1711 genes; best 5 terms/source)

source	term_name	term_id	adjusted_p_value
GO:MF	protein domain specific binding	GO:0019904	3.23E-11
GO:MF	kinase activity	GO:0016301	2.29E-07
GO:MF	ion binding	GO:0043167	3.74E-07
GO:MF	photoreceptor activity	GO:0009881	1.49071E-06
GO:MF	adenyl nucleotide binding	GO:0030554	2.52347E-06
GO:BP	response to abiotic stimulus	GO:0009628	6.79E-29
GO:BP	response to stimulus	GO:0050896	3.45E-22
GO:BP	response to chemical	GO:0042221	2.16E-17
GO:BP	response to stress	GO:0006950	3.97E-15
GO:BP	response to light stimulus	GO:0009416	4.75E-14
GO:CC	chloroplast	GO:0009507	2.68E-39
GO:CC	plastid	GO:0009536	3.22E-39
GO:CC	chloroplast stroma	GO:0009570	4.09E-19
GO:CC	plastid stroma	GO:0009532	9.09E-19
GO:CC	cytoplasm	GO:0005737	5.69E-18

NT; Col-0 vs tfIIs-1; higher expression in tfIIs-1 (1879 genes; best 5 terms/source)

source	term_name	term_id	adjusted_p_value
GO:MF	structural constituent of ribosome	GO:0003735	3.04E-104
GO:MF	structural molecule activity	GO:0005198	1.79E-94
GO:MF	mRNA binding	GO:0003729	7.96E-39
GO:MF	copper ion binding	GO:0005507	1.56E-26
GO:MF	oxidoreductase activity	GO:0016491	2.14E-23
GO:BP	organonitrogen compound biosynthetic process	GO:1901566	1.09E-62
GO:BP	peptide metabolic process	GO:0006518	4.60E-54
GO:BP	cellular amide metabolic process	GO:0043603	8.36E-54
GO:BP	translation	GO:0006412	2.79E-49
GO:BP	peptide biosynthetic process	GO:0043043	7.43E-49
GO:CC	cytosolic ribosome	GO:0022626	3.17E-123
GO:CC	ribosomal subunit	GO:0044391	2.45E-99
GO:CC	ribosome	GO:0005840	1.31E-98
GO:CC	cytosol	GO:0005829	1.17E-87
GO:CC	polysomal ribosome	GO:0042788	3.15E-72

B

1h; Col-0 vs tfIIs-1; higher expression in Col-0 (1756 genes; best 5 terms/source)

source	term_name	term_id	adjusted_p_value
GO:MF	unfolded protein binding	GO:0051082	2.76E-14
GO:MF	protein binding	GO:0005515	1.29E-09
GO:MF	heat shock protein binding	GO:0031072	5.04E-09
GO:MF	misfolded protein binding	GO:0051787	0.00017
GO:MF	protein self-association	GO:0043621	0.000178
GO:BP	response to abiotic stimulus	GO:0009628	6.31E-46
GO:BP	response to chemical	GO:0042221	8.43E-45
GO:BP	response to stress	GO:0006950	1.80E-41
GO:BP	response to temperature stimulus	GO:0009266	1.99E-33
GO:BP	response to stimulus	GO:0050896	6.42E-33
GO:CC	cytoplasm	GO:0005737	4.71E-34
GO:CC	intracellular anatomical structure	GO:0005622	8.46E-28
GO:CC	vacuole	GO:0005773	3.20E-19
GO:CC	endomembrane system	GO:0012505	4.66E-18
GO:CC	intracel. membrane-bounded organelle	GO:0043231	4.33E-17

1h; Col-0 vs tfIIs-1; higher expression in tfIIs-1 (1102 genes; best 5 terms/source)

source	term_name	term_id	adjusted_p_value
GO:MF	mRNA binding	GO:0003729	4.56E-20
GO:MF	RNA binding	GO:0003723	9.31E-10
GO:MF	nucleic acid binding	GO:0003676	4.00E-09
GO:MF	structural molecule activity	GO:0005198	7.93E-07
GO:MF	structural constituent of ribosome	GO:0003735	1.14E-05
GO:BP	response to abiotic stimulus	GO:0009628	3.18E-21
GO:BP	biosynthetic process	GO:0009058	1.82E-18
GO:BP	photosynthesis	GO:0015979	1.95E-18
GO:BP	organic substance biosynthetic process	GO:1901576	2.89E-17
GO:BP	response to light stimulus	GO:0009416	2.30E-16
GO:CC	chloroplast	GO:0009507	3.95E-58
GO:CC	plastid	GO:0009536	2.53E-55
GO:CC	cytoplasm	GO:0005737	4.25E-37
GO:CC	plastid envelope	GO:0009526	2.84E-36
GO:CC	intracellular anatomical structure	GO:0005622	3.49E-36

C

1d; Col-0 vs tfIIs-1; higher expression in Col-0 (2939 genes; best 5 terms/source)

source	term_name	term_id	adjusted_p_value
GO:MF	nucleic acid binding	GO:0003676	6.16E-16
GO:MF	catalytic activity, acting on RNA	GO:0140098	2.32E-15
GO:MF	protein binding	GO:0005515	1.31E-13
GO:MF	binding	GO:0005488	5.96E-13
GO:MF	RNA binding	GO:0003723	8.11E-13
GO:BP	nitrogen compound metabolic proces	GO:0006807	4.97E-44
GO:BP	organic substance metabolic process	GO:0071704	1.56E-42
GO:BP	primary metabolic process	GO:0044238	4.14E-42
GO:BP	nucleobase-containing compound	GO:0006139	1.25E-41
GO:BP	macromolecule metabolic process	GO:0043170	7.90E-41
GO:CC	intracellular anatomical structure	GO:0005622	1.39E-89
GO:CC	intracellular organelle	GO:0043229	2.78E-59
GO:CC	organelle	GO:0043226	1.72E-57
GO:CC	intracel membrane-bound organelle	GO:0043231	3.72E-53
GO:CC	membrane-bounded organelle	GO:0043227	2.73E-51

1d; Col-0 vs tfIIs-1; higher expression in tfIIs-1 (4259 genes; best 5 terms/source)

source	term_name	term_id	adjusted_p_value
GO:MF	mRNA binding	GO:0003729	8.27E-98
GO:MF	RNA binding	GO:0003723	7.53E-56
GO:MF	structural molecule activity	GO:0005198	2.87E-53
GO:MF	structural constituent of ribosome	GO:0003735	3.04E-52
GO:MF	protein domain specific binding	GO:0019904	5.54E-25
GO:BP	response to abiotic stimulus	GO:0009628	1.84E-45
GO:BP	generation of precursor metabolites and energy	GO:0006091	2.38E-45
GO:BP	photosynthesis	GO:0015979	2.29E-43
GO:BP	organonitrogen compound biosynthetic process	GO:1901566	1.91E-40
GO:BP	peptide metabolic process	GO:0006518	8.06E-40
GO:CC	cytoplasm	GO:0005737	1.19E-214
GO:CC	cytosol	GO:0005829	7.19E-153
GO:CC	plastid	GO:0009536	8.71E-148
GO:CC	chloroplast	GO:0009507	1.29E-119
GO:CC	organelle envelope	GO:0031967	4.39E-114

D

1d+rec; Col-0 vs tfIIs-1; higher expression in Col-0 (1042 genes; best 5 terms/source)

source	term_name	term_id	adjusted_p_value
GO:MF	protein domain specific binding	GO:0019904	1.57E-19
GO:MF	chlorophyll binding	GO:0016168	0.000131
GO:MF	tetrapyrrole binding	GO:0046906	0.000224
GO:MF	catalytic activity	GO:0003824	0.002551
GO:MF	water channel activity	GO:0015250	0.002571
GO:BP	photosynthesis	GO:0015979	1.84E-23
GO:BP	response to stimulus	GO:0050896	3.51E-16
GO:BP	response to abiotic stimulus	GO:0009628	1.06E-14
GO:BP	response to stress	GO:0006950	3.12E-14
GO:BP	response to external stimulus	GO:0009605	1.23E-13
GO:CC	chloroplast	GO:0009507	1.02E-48
GO:CC	plastid	GO:0009536	3.07E-47
GO:CC	thylakoid	GO:0009579	2.68E-43
GO:CC	chloroplast thylakoid	GO:0009534	1.44E-42
GO:CC	plastid thylakoid	GO:0031976	1.74E-42

1d+rec; Col-0 vs tfIIs-1; higher expression in tfIIs-1 (995 genes; best 5 terms/source)

source	term_name	term_id	adjusted_p_value
GO:MF	structural constituent of ribosome	GO:0003735	1.41E-20
GO:MF	oxidoreductase activity	GO:0016491	1.43E-19
GO:MF	structural molecule activity	GO:0005198	2.74E-19
GO:MF	unfolded protein binding	GO:0051082	2.80E-15
GO:MF	mRNA binding	GO:0003729	8.11E-11
GO:BP	response to chemical	GO:0042221	1.75E-40
GO:BP	response to inorganic substance	GO:0010035	7.79E-33
GO:BP	response to stimulus	GO:0050896	1.63E-32
GO:BP	response to cadmium ion	GO:0046686	4.01E-24
GO:BP	response to abiotic stimulus	GO:0009628	4.44E-22
GO:CC	cytosol	GO:0005829	9.08E-38
GO:CC	cytosolic ribosome	GO:0022626	9.54E-30
GO:CC	cytoplasm	GO:0005737	1.44E-28
GO:CC	external encapsulating structure	GO:0030312	1.95E-28
GO:CC	cell wall	GO:0005618	3.54E-28

Supplementary figure 10: Gene ontology (GO) term analysis of differentially expressing genes (DEGs) between Col-0 and *tfIIs-1* at different heat stress treatments. Numbers of DEGs in Col-0 and *tfIIs-1* are shown above; best 5 terms/source of molecular function (MF), biological process (BP), cell compartment (CC) are listed; terms ID numbers and adjusted significance p values are shown for each.

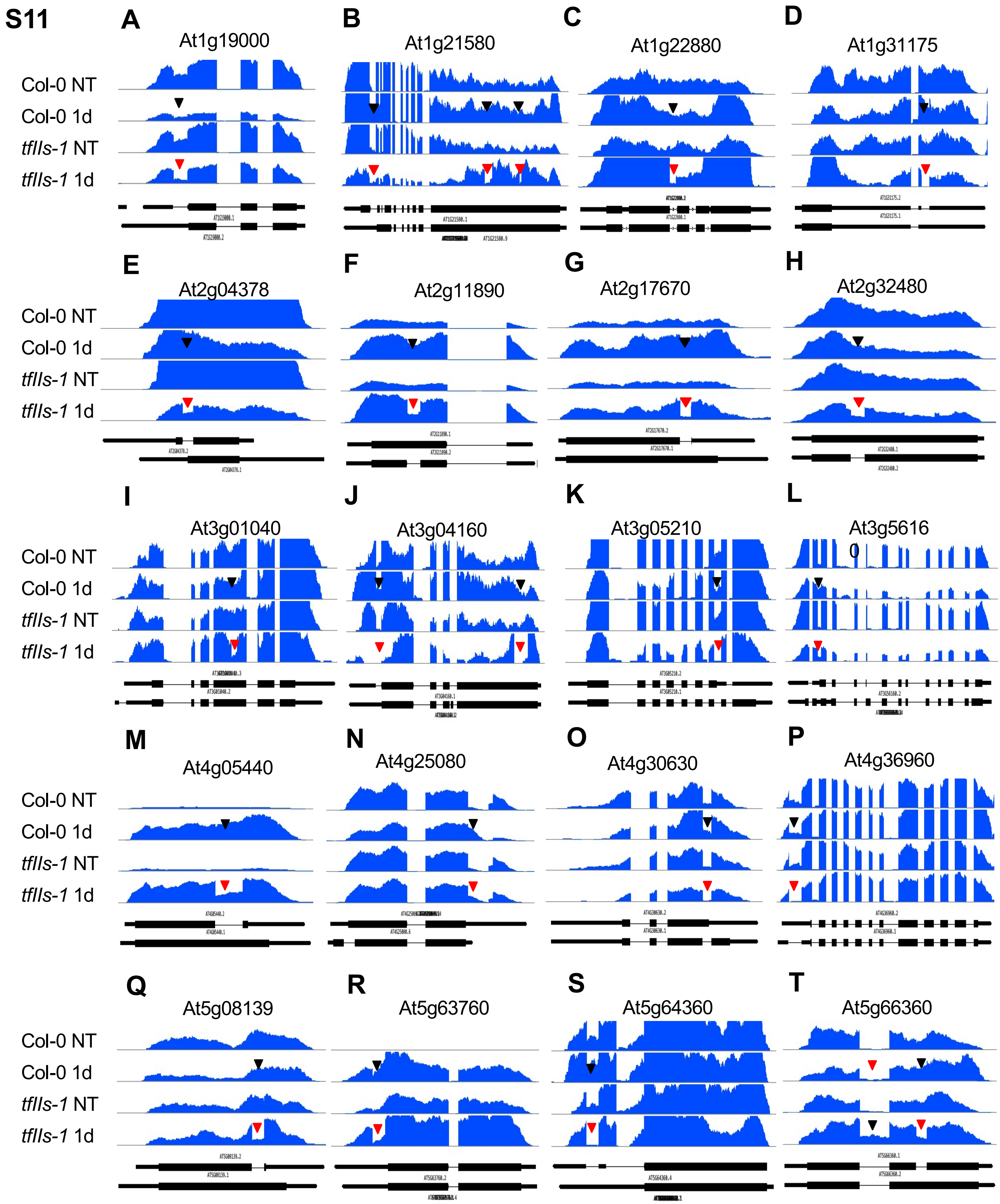

Supplementary figure 11: Alternative splicing (AS) event changes during heat treatment in Col-0 and *tfIIs-1* plants. Genome browser images of transcriptome read tracks on selected loci with altered AS events (black and red triangles point to regions having altered isoform expression patterns). Genotypes and heat treatment conditions are shown on the left, schematic of AS variant at the bottom.

S12

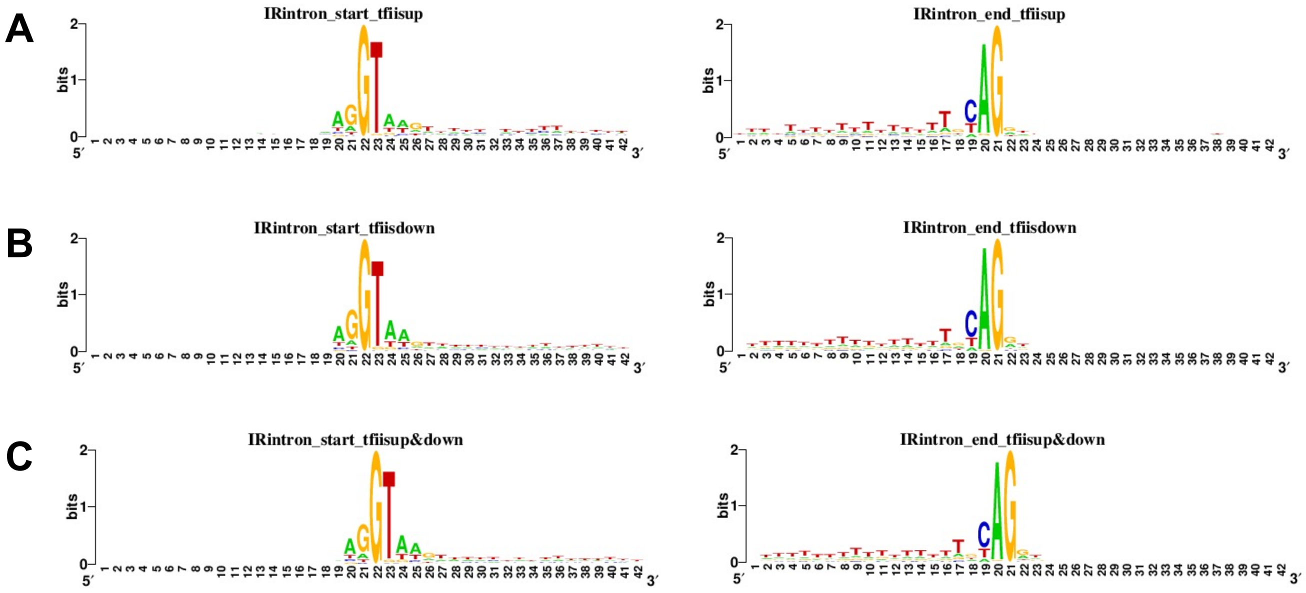

Supplementary figure 12: 5' or 3' splice sites of alternatively spliced introns (intron retention events (IR) in *tfIIs-1* mutant during heat. WebLogo of donor (left) and acceptor (right) splice sites originating from genes differentially higher expression (A), differentially down-regulated (B) combined (C) in *tfIIs-1* mutant plants; both donor and acceptor sites are very similar to *Arabidopsis* whole genome U2-type intron consensus sites (not shown); X-axes correspond to nucleotides surrounding donor and acceptor splice site, position 21 corresponds to last nucleotide of upstream exon (left), while position 22 corresponds to first nucleotide in the down-stream exon (right); Y-axes represent information contents in alignment and nucleotide frequency (bits).

S13

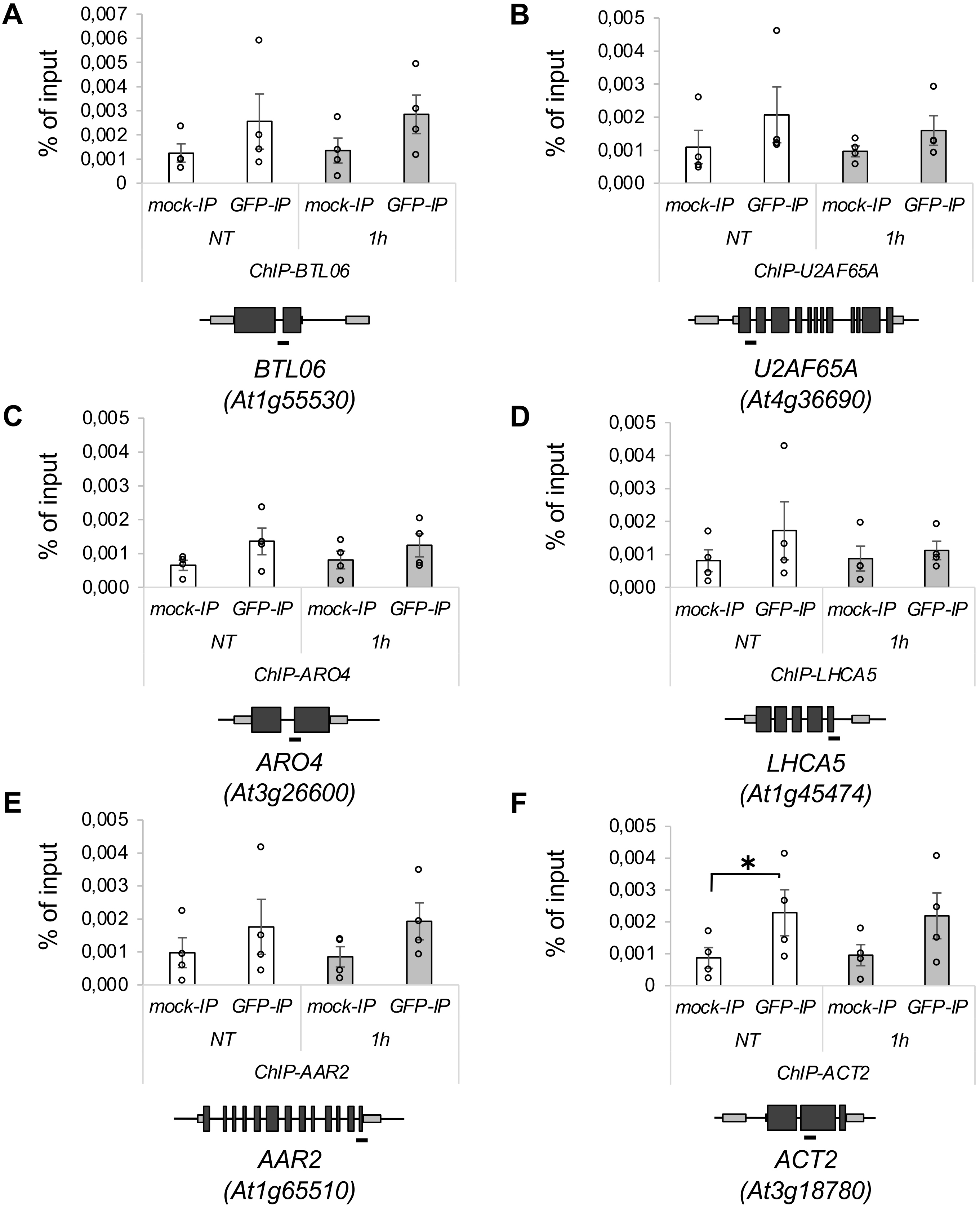

Supplementary figure S13: TFIIS association with alternatively spliced (AS) Pol II-transcribed loci. Chromatin immunoprecipitation – qPCR analysis of GSy-TFIIS binding to (A) BTL06, (B) U2AF65A, (C) ARO4, (D) LHCA5, (E) AAR2 and (F) ACT2 genes. Location of qPCR amplicons are shown as horizontal segments below the schematic of each locus (not to scale); bars represent standard errors based on at least three biological replicates; p values based on two-tailed Student's t-test (*p<0.05, **p<0.01, ***p<0.001).

S14

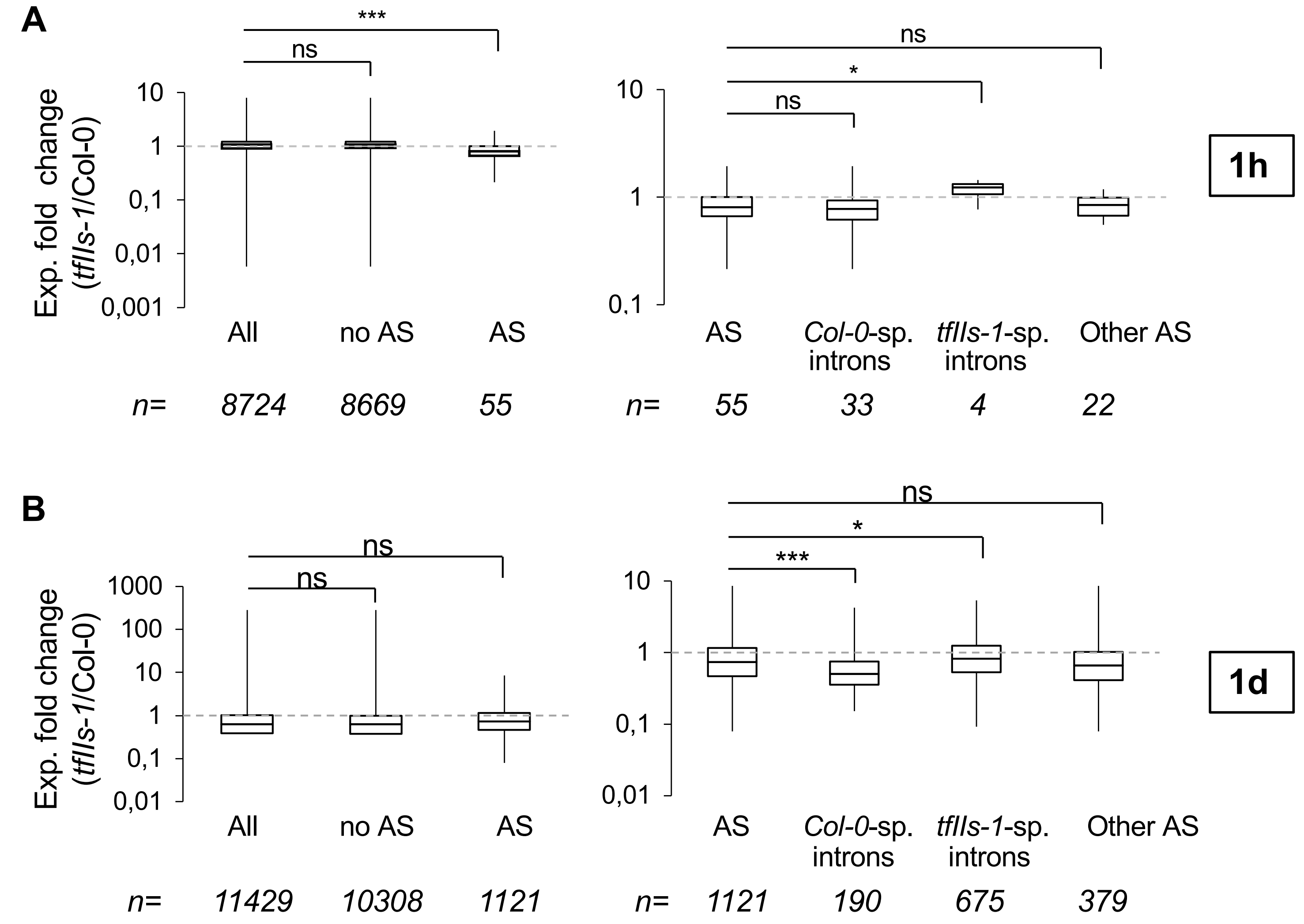

Supplementary figure 14: TFIIS-dependent IR events are correlated with expression level at 1 hour (A) and 1 day (B) heat treatment. All genes (all, FPKM ≧ 5) or diverse AS gene groups (differing between *tfIIs-1* and Col-0) are shown on X axis, genes with unchanged splicing pattern (no AS), *Col-0*-specific intron (Col-0-sp. introns; e.g. introns are more strongly spliced out in Col-0 *plants*), *tfIIs-1*-specific intron (*tfIIs-1*-sp. introns; e.g. introns are more strongly spliced out in *tfIIs-1 plants*), other alternative splicing events (other AS); number of genes (n) is shown below each group. Expression fold change (*tfIIs-1*/Col-0 ratio) are shown on y axis. p values based on two-tailed Student's t-test (✱p<0.05, ✱✱p<0.01, ✱✱✱p<0.001).

Supplementary figure 15: HSR pathway is lagging behind in *tfIIs-1* mutant plants. Normalized FPKM value changes of selected heat shock (HSP) transcripts during heat-treated time-series in Col-0 and *tfIIs-1* plants; early-induced and late-induced gene transcripts are separated in the upper and lower part of the table, respectively; genotypes and treatments are shown at the top, gene names on the left; expression values and colour codes are shown for each by line; standard error values calculated based on four biological replicates is shown below in brackets.

▼

S15

Low High

		NT		1h		1d		1d+rec	
		Col-0	tflls-1	Col-0	tflls-1	Col-0	tflls-1	Col-0	tflls-1
Early HS transcripts	HSP15.7	1.2 (0.6)	1.1 (0.2)	910.7 (25.3)	876.1 (50.0)	33.2 (9.2)	23.3 (3.6)	4.8 (0.6)	5.7 (1.4)
	HSP17.4A	1.0 (0.7)	1.6 (1.2)	8218.0 (234.4)	7062.4 (288.8)	3926.7 (755.6)	5821.4 (562.7)	25.4 (4.7)	66.2 (16.8)
	HSP17.4B	2.6 (0.9)	3.7 (0.3)	2807.6 (97.2)	2284.2 (124.9)	142.0 (38.7)	79.9 (10.5)	2.8 (0.3)	2.9 (1.0)
	HSP17.6	0.6 (0.6)	1.9 (1.6)	5540.8 (107.0)	5806.0 (101.9)	2418.4 (690.9)	2292.6 (547.0)	5.1 (0.9)	16.8 (5.2)
	HSP17.6A	1.1 (0.7)	1.5 (0.5)	4718.3 (163.1)	3279.7 (35.5)	83.3 (14.6)	115.6 (10.5)	1.2 (0.4)	1.5 (0.6)
	HSP17.6B	3.0 (1.3)	4.8 (1.2)	4256.1 (125.6)	3887.4 (147.8)	1090.3 (139.3)	3556.8 (420.6)	26.0 (5.4)	81.4 (9.2)
	HSP17.6C	0.2 (0.3)	1.5 (1.4)	8287.3 (128.1)	6825.6 (193.3)	2287.7 (625.6)	5308.6 (946.7)	20.1 (6.5)	40.6 (5.1)
	HSP17.8	2.0 (0.7)	4.5 (1.7)	10132.7 (349.3)	6815.5 (208.3)	537.4 (92.6)	899.2 (86.4)	3.1 (0.4)	5.5 (1.6)
	HSP18.1	0.9 (0.7)	2.3 (1.5)	5370.4 (143.2)	4531.9 (189.0)	3280.8 (807.8)	14071.4 (2107.9)	252.0 (71.8)	498.9 (81.9)
	HSP18.5	18.3 (1.1)	13.7 (1.3)	1031.1 (42.2)	767.2 (34.0)	362.5 (92.2)	775.6 (98.7)	29.0 (4.2)	25.9 (4.9)
	HSP22.0	0.3 (0.6)	0.3 (0.5)	3073.4 (48.9)	2590.9 (69.6)	2652.1 (549.4)	3719.2 (473.9)	10.2 (2.0)	24.7 (5.4)
	HSP23.5	2.0 (0.5)	4.1 (0.8)	1922.3 (66.6)	1913.6 (36.3)	441.6 (54.7)	462.4 (21.0)	1.4 (0.4)	4.4 (1.0)
	HSP23.6	0.1 (0.1)	0.3 (0.4)	5079.7 (57.4)	4564.7 (128.1)	1066.4 (212.4)	797.1 (90.1)	1.2 (0.4)	4.1 (1.0)
	HSP26.5	0.0 (0.1)	0.2 (0.2)	786.7 (85.9)	537.5 (27.3)	71.1 (8.1)	35.0 (5.4)	0.6 (0.1)	1.3 (0.5)
	HSP70-10	11.2 (2.2)	19.3 (3.0)	1488.2 (40.6)	1186.1 (39.5)	462.6 (101.6)	516.6 (64.8)	15.4 (1.5)	25.9 (1.4)
	HSP70-3	70.6 (6.9)	91.9 (7.5)	3390.4 (102.4)	2750.7 (108.1)	354.2 (26.7)	350.2 (44.5)	38.1 (3.4)	50.2 (2.8)
	HSP70-4	8.0 (1.5)	13.6 (2.5)	6449.6 (306.2)	4933.8 (220)	1573.7 (185.9)	1576.9 (106.8)	10.6 (1.7)	21.5 (3.3)
	HSP70-5	1.8 (0.6)	2.0 (0.7)	5037.6 (211.9)	4313.2 (149.7)	228.1 (30.3)	480.8 (53.2)	3.1 (0.4)	3.9 (0.9)
	HSP70-8	1.9 (0.2)	1.3 (0.3)	1037.9 (29.1)	959.1 (5.0)	134.4 (21.8)	260.9 (27.1)	3.1 (0.4)	4.6 (0.8)
	HSP81-2	51.1 (6.3)	71.4 (8.3)	3418.8 (139.9)	1873.0 (41.9)	556.9 (54.38)	521.3 (33.5)	38.2 (4.2)	50.4 (4.6)
	HSP90-1	3.5 (1.3)	4.2 (1.4)	5342.5 (337.1)	3673.0 (190.1)	790.8 (184.4)	748.1 (65.3)	4.1 (0.6)	7.7 (1.5)
	HSP90-3	57.0 (6.1)	73.8 (8.9)	1185.9 (69.4)	1035.0 (34.6)	271.2 (46.35)	159.4 (13.9)	31.7 (4.0)	39.6 (5.4)
	HSP101	2.9 (0.5)	3.8 (0.3)	4743.7 (218.6)	3889.1 (183.2)	198.9 (27.2)	242.7 (22.5)	2.4 (0.4)	2.4 (0.6)
Late HS transcripts	HSP14.7	0.6 (0.8)	0.4 (0.4)	0.2 (0.2)	0.4 (0.3)	78.8 (18.1)	10.0 (2.2)	1.2 (0.7)	0.6 (0.5)
	HSP21	0.1 (0.2)	0.0 (0.1)	1275.1 (51.0)	768.2 (38.0)	2424.5 (235.7)	1247.0 (78.9)	3.9 (1.3)	13.2 (5.2)
	HSP60-2	10.6 (1.5)	18.5 (1.8)	8.0 (0.9)	9.7 (1.1)	64.8 (21.5)	38.9 (8.0)	15.2 (2.2)	20.2 (1.0)
	HSP60-3A	7.8 (1.0)	12.0 (1.3)	18.2 (2.9)	11.3 (1.0)	180.4 (46.5)	131.7 (22.1)	8.8 (1.0)	14.1 (1.4)
	HSP70-14	24.8 (0.6)	33.1 (2.0)	30.9 (1.2)	27.8 (0.2)	80.1 (13.8)	52.6 (7.9)	31.4 (1.3)	35.3 (2.3)
	HSP70-16	9.9 (0.7)	10.3 (1.1)	40.1 (5.2)	20.0 (1.4)	45.4 (15.4)	20.1 (4.6)	12.0 (0.4)	13.0 (0.4)
	HSP70-6	68.8 (5.6)	81.5 (4.4)	274.2 (7.9)	279.7 (5.8)	569.6 (111.0)	335.8 (46.2)	95.3 (4.9)	125.7 (2.8)
	HSP70-7	51.6 (4.5)	69.1 (5.2)	175.6 (3.7)	182.2 (6.4)	395.8 (76.2)	163.8 (23.0)	55.9 (4.3)	60.2 (4.0)
	HSP70-9	21.9 (1.8)	31.4 (4.7)	24.8 (2.2)	21.9 (1.2)	211.3 (46.54)	280.2 (31.4)	28.1 (1.9)	42.6 (4.3)
	HSP90-4	8.8 (1.4)	11.3 (0.7)	11.5 (0.5)	18.8 (1.2)	27.0 (4.3)	22.5 (2.5)	7.5 (1.1)	9.5 (0.9)
	HSP90-5	36.4 (3.1)	48.6 (4.9)	123.7 (7.2)	106.5 (3.1)	617.9 (216.2)	214.1 (42.4)	43.7 (4.3)	64.4 (3.4)
	HSP90-6	15.5 (1.9)	16.8 (2.5)	86.2 (5.6)	72.0 (3.7)	261.3 (71.7)	133.4 (19.5)	11.6 (2.0)	17.1 (1.6)

S16

Low High

	NT		1h		1d		1d+rec	
	**Col-0**	***tfIIs-1***	**Col-0**	***tfIIs-1***	**Col-0**	***tfIIs-1***	**Col-0**	***tfIIs-1***
**PSAD1**	1849.0 (29.8)	1634.6 (40.8)	1256.6 (27.8)	1486.4 (73.3)	178.0 (13.8)	491.2 (23.5)	1391.0 (85.9)	1110.8 (70.9)
**PSAD2**	982.7 (37.8)	854.7 (51.6)	633.9 (15.2)	848.9 (52.4)	34.3 (6.3)	173.5 (4.2)	631.7 (61.0)	545.3 (39.6)
**PSAE1**	4500.0 (456.0)	4490.1 (502.6)	3152.5 (353.6)	3692.9 (343.4)	753.7 (137.2)	2239.1 (217.8)	3235.8 (329.5)	2879.6 (238.8)
**PSAE2**	1111.6 (92.4)	855.0 (80.3)	747.5 (19.7)	744.0 (30.2)	467.0 (95.3)	617.0 (51.9)	1001.1 (49.4)	801.1 (40.2)
**PSAF**	3219.3 (134.3)	2830.9 (183.5)	2483.5 (50.1)	2556.6 (71.6)	584.2 (109.6)	1642.5 (60.5)	2181.9 (139.8)	1747.4 (129.8)
**PSAG**	3408.8 (129.0)	2798.0 (170.4)	2952.2 (92.2)	3041.2 (99.4)	924.9 (273.7)	1667.3 (124.6)	2457.4 (123.5)	2056.7 (153.9)
**PSAH1**	769.4 (33.9)	736.1 (39.3)	581.7 (14.5)	702.6 (18.3)	208.7 (62.7)	742.1 (14.6)	632.5 (15.0)	567.3 (39.0)
**PSAH2**	1351.6 (24.8)	1410.9 (66.5)	1079.5 (35.8)	1359.1 (33.9)	101.8 (23.8)	748.9 (49.9)	1056.1 (75.7)	990.0 (56.2)
**PSAK**	2144.8 (16.1)	2031.8 (154.6)	1701.3 (47.1)	2054.2 (12.9)	233.6 (77.3)	1064.0 (157.5)	1722.2 (124.6)	1409.8 (78.6)
**PSAL**	3339.3 (110.6)	2997.9 (131.5)	2570.6 (91.0)	2868.0 (97.5)	568.0 (140.4)	2038.5 (104.0)	2460.8 (146.9)	2043.1 (151.4)
**PSAN**	1930.1 (61.6)	1700.5 (149.1)	1285.2 (24.0)	1403.8 (26.4)	69.0 (10.6)	185.8 (12.1)	1525.2 (100.3)	1207.4 (52.2)
**PSAO**	3458.8 (249.6)	2924.5 (190.8)	2321.5 (40.8)	2740.1 (102.6)	155.3 (27.7)	763.6 (49.1)	2709.0 (172.0)	2206.9 (160.8)
**PSB27-1**	305.5 (11.0)	352.8 (35.8)	276.7 (14.9)	352.1 (13.1)	146.3 (25.5)	266.4 (11.5)	295.1 (12.6)	262.4 (15.1)
**PSB28**	83.5 (6.9)	109.9 (6.7)	75.8 (1.9)	95.1 (11.6)	45.4 (14.1)	62.6 (6.4)	109.5 (6.9)	105.3 (3.2)
**PSBO1**	3472.2 (207.0)	3079.1 (296.5)	2362.5 (86.0)	2659.3 (145.0)	955.7 (67.6)	2150.2 (46.4)	2495.8 (126.3)	2135.6 (145.4)
**PSBO2**	873.0 (43.7)	946.5 (23.5)	787.8 (36.6)	1102.9 (50.4)	189.4 (17.3)	741.4 (26.7)	795.4 (43.1)	818.5 (66.8)
**PSBP1**	2721.5 (90.4)	2743.0 (166.6)	1997.7 (55.4)	2303.1 (114.7)	925.8 (46.1)	1718.1 (50.8)	2282.1 (108.0)	2013.5 (129.4)
**PSBQ1**	1330.4 (40.6)	1532.0 (78.9)	1322.2 (35.0)	1576.7 (65.5)	299.2 (28.4)	1119.1 (59.3)	1303.7 (75.5)	1193.8 (53.0)
**PSBQ2**	1649.6 (41.6)	1386.9 (71.3)	1238.2 (35.8)	1342.8 (65.1)	225.9 (32.4)	785.6 (13.2)	1446.1 (105.6)	1201.3 (94.5)
**PSBTN**	1763.8 (56.0)	1925.2 (143.8)	1089.5 (61.0)	1315.4 (94.2)	199.7 (42.4)	830.9 (23.0)	1663.9 (66.1)	1475.3 (146.2)
**PSBW**	2295.9 (48.4)	2130.5 (129.8)	1458.9 (36.5)	1910.5 (34.1)	762.1 (182.4)	1476.9 (112.1)	1845.8 (109.3)	1601.6 (83.6)
**PSBX**	2192.4 (98.9)	2149.2 (148.3)	1367.0 (51.7)	1713.9 (68.4)	351.8 (104.8)	948.3 (122.1)	1900.4 (134.6)	1684.1 (97.3)
**PSBY**	747.2 (41.3)	825.3 (71.7)	691.3 (26.3)	895.4 (50.9)	97.1 (25.4)	489.3 (32.4)	629.7 (42.5)	617.4 (64.4)

Supplementary figure 16: Photosynthesis-related transcripts are altered in the absence of TFIIS. (A) Normalized FPKM value changes of photosystem PSII (PSA) and PSI (PSB) component transcripts during heat-treated time-series in Col-0 and *tfIIs-1* plants; genes are shown on the left; expression values and colour codes are shown for each by line; standard errors calculated based on four biological replicates is shown below in brackets.

S17

Low — High

	NT		1h		1d		1d+rec	
	**Col-0**	***tfIIs-1***	**Col-0**	***tfIIs-1***	**Col-0**	***tfIIs-1***	**Col-0**	***tfIIs-1***
**LHB1B1**	7455.9 (613.2)	5444.4 (528.5)	4263.8 (393.2)	4326.1 (212.1)	343.2 (119.7)	1212.2 (154.7)	3464.5 (339.1)	1786.3 (285.0)
**LHB1B2**	17070.2 (802.8)	13502.0 (1266.1)	14566.1 (419.1)	15105.1 (772.2)	2606.7 (949.5)	9314.8 (1133.1)	10284.7 (661.5)	7523.9 (537.1)
**LHCA1**	5726.7 (270.1)	4926.2 (300.7)	3879.7 (116.3)	4303.0 (104.8)	2046.3 (271.6)	3543.8 (213.0)	3452.2 (217.8)	2982.7 (214.3)
**LHCA2**	3882.3 (221.9)	3234.0 (176.6)	3148.0 (92.4)	3318.6 (39.6)	1146.8 (176.9)	2801.1 (127.4)	2632.9 (187.5)	2079.4 (141.1)
**LHCA3**	3330.7 (151.0)	2827.9 (185.3)	3001.9 (37.9)	3246.5 (99.4)	993.9 (160.2)	2534.1 (84.6)	2262.7 (139.7)	1922.6 (142.1)
**LHCA4**	5458.8 (178.6)	4705.4 (270.7)	4640.4 (209.4)	5000.4 (121.7)	312.5 (38.0)	1685.7 (160.2)	2984.5 (217.3)	2415.8 (131.3)
**LHCA5**	149.1 (2.1)	148 (4.7)	123.1 (7.6)	148.4 (9.7)	32.2 (2.3)	52.4 (2.4)	128.1 (9.5)	128.1 (9.2)
**LHCB1.1**	4899.3 (357.8)	5486.1 (184.0)	3814.6 (226.6)	4775.1 (355.1)	179.1 (33.9)	1540.9 (139.6)	1973.6 (347.8)	2012.3 (152.3)
**LHCB1.1**	5607.3 (408.3)	5685.4 (148.4)	4701.9 (336.9)	4920.9 (357.3)	1539.1 (186.8)	3392.5 (148.1)	3342.5 (287.4)	3002.3 (263.0)
**LHCB1.3**	20942.8 (1441.7)	17020.5 (873.4)	14233.3 (681.0)	14576.6 (576.6)	1684.1 (266.9)	6830.9 (368.3)	15407.6 (758.5)	12361.2 (871.9)
**LHCB2.1**	2897.2 (152.7)	2844.3 (146.9)	2246.1 (84.7)	2978.2 (196.2)	24.3 (6.4)	78.3 (6.5)	979.5 (124.9)	978.9 (98.4)
**LHCB2.2**	2690.1 (151.9)	2591.1 (72.7)	2065.1 (60.3)	2597.9 (188.2)	22.7 (6.2)	101.2 (4.5)	1141.1 (129.1)	1169.9 (119.4)
**LHCB2.4**	785.1 (69.0)	891.7 (68.5)	691.6 (35.7)	1029.7 (99.2)	0.9 (0.5)	7.6 (1.3)	230.6 (54.9)	208.0 (33.6)
**LHCB3**	4085.0 (117.1)	3991.4 (404.2)	2879.9 (85.6)	3553.5 (148.5)	214.8 (49.3)	1351.8 (105.2)	2587.1 (289.4)	2295.5 (196.7)
**LHCB4.1**	3730.5 (170.6)	3221.2 (114.3)	2736.3 (144.1)	2920.8 (181.8)	1268.1 (20.7)	2003.3 (83.9)	2684.0 (173.9)	2234.3 (169.6)
**LHCB4.2**	1886.7 (93.0)	1618.6 (93.2)	1271.0 (73.4)	1516.5 (69.0)	89.5 (6.4)	339.5 (38.4)	956.7 (80.9)	812.2 (97.0)
**LHCB5**	4956.1 (107.1)	4849.5 (249.3)	3386.9 (113.7)	4161.4 (198.2)	1451.0 (152.0)	2556.7 (87.7)	3554.3 (207.3)	3093.4 (176.0)
**LHCB6**	5135.4 (289.5)	5276.0 (320.5)	4484.5 (139.8)	5113.8 (164.2)	542.3 (110.5)	1270.5 (35.2)	3881.4 (411.7)	3477.9 (343.8)

Supplementary figure 17: Photosynthesis-related transcripts are altered in the absence of TFIIS. (A) Normalized FPKM value changes of Light Harvesting Complex component transcripts (for photosystem PSII (LHCA) and PSI (LHCB)) during heat-treated time-series in Col-0 and *tfIIs-1* plants; genes are shown on the left; expression values and colour codes are shown for each by line; standard errors calculated based on four biological replicates is shown below in brackets.

S18

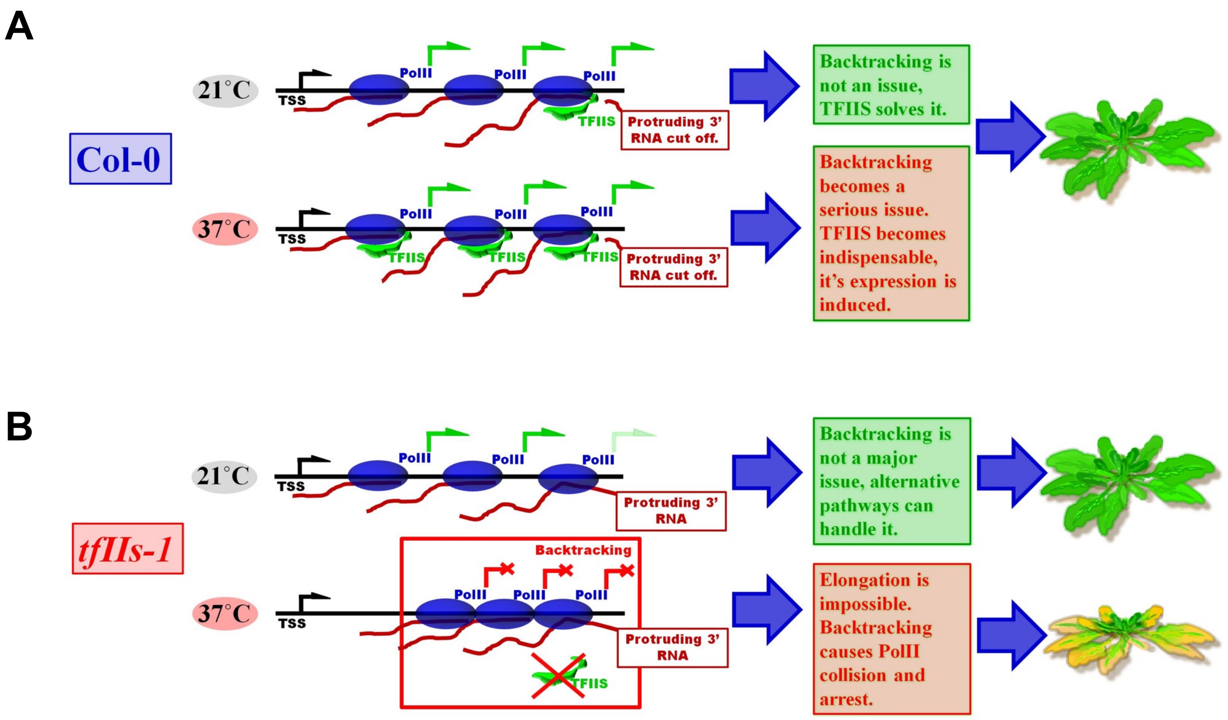

Supplementary figure 18: Working model for TFIIS roles during heat stress adaptation. (A) In Col-0 plants TFIIS interacts with RNAPII to enable efficient transcription; arrested complexes are efficiently resolved; During heat stress conditions HsfA1s *trans* factors transcriptionally induce TFIIS to increase efficiency of RNAPII arrest resolution and transcriptional output: plants are heat tolerant. (B) In the absence of TFIIS factor (in *tfIIs* mutants) arrested RNAPII events are surpassed by RNAPII intrinsic activity and potentially resolved through alternative routes; plant development is unaffected. During heat stress the absence of TFIIS cannot be compensated due to either (i) increased amounts of arrest/backtracking events, (ii) the decreased intrinsic nuclease activity of RNAPII or (iii) overloaded alternative mechanisms directed to resolve RNAPII arrested/backtracking events; this leads to delayed and improper transcriptional output (slow induction of HS transcripts and altered splicing) and indirectly causes lethality.
