# Supplementary material for: Elongation factor TFIIS is essential for heat stress adaptation in plants
Source: Nucleic Acids Res. 2022 Jan 31;50(4):1927–50. doi: 10.1093/nar/gkac020 (PMC8886746; doi:10.1093/nar/gkac020)
Supplement: gkac020_Supplemental_Files [file gkac020_supplemental_files.zip › Supplementary Table Legends_20211211.pdf]

Supplementary Table S1: DNA oligonucleotide sequences used in the study.

Supplementary Table S2: RNA sequencing data (FPKM values) of all genes in Col-0 and *tfIIs-1* mutant plants under control (NT), heat stress treatment (1h and 1d, or post-stress (1d+rec) conditions and DEGs (foldchange higher than 2 and $p \leq 0.05$).

Supplementary Table S3: Genomic regions, loci and types of differencial alternative splicing (AS) events in Col-0 and *tfIIs-1* mutant plants under control (NT), heat stress treatment (1h and 1d), or post-stress (1d+rec) conditions.

Supplementary Table S4: Gene ontology (GO) term analysis of alternatively spliced gene/transcript groups detected in Col-0 and *tfIIs-1* mutant plants under heat stress treatment (1h and 1d) conditions.
